# Corrosion Response of Steel to Penetration of Chlorides in DC-Treated Hardened Portland Cement Mortar

**DOI:** 10.3390/ma18143365

**Published:** 2025-07-17

**Authors:** Milan Kouřil, Jan Saksa, Vojtěch Hybášek, Ivona Sedlářová, Jiří Němeček, Martina Kohoutková, Jiří Němeček

**Affiliations:** 1 Department of Metals and Corrosion Engineering, University of Chemistry and Technology in Prague, Technická 5, 166 28 Prague, Czech Republichybasekv@vscht.cz (V.H.); 2 Department of Inorganic Technology, University of Chemistry and Technology in Prague, Technická 5, 166 28 Prague, Czech Republic; sedlaroi@vscht.cz; 3 Department of Mechanics, Faculty of Civil Engineering, Czech Technical University, Thákurova 7, 166 29 Prague, Czech Republic; jiri.nemecek.1@fsv.cvut.cz (J.N.); jiri.nemecek@fsv.cvut.cz (J.N.); 4 Central Laboratories, University of Chemistry and Technology in Prague, Technická 5, 166 28 Prague, Czech Republic; kohoutkm@vscht.cz

**Keywords:** electric current, mortar, porosity, chloride penetration, corrosion

## Abstract

Electrochemical treatment by means of direct current (DC) is usually used as a measure for steel rebar corrosion protection, e.g., cathodic protection (CP), electrochemical chloride extraction (ECE), and re-alkalization (RA). However, the passage of an electrical charge through the pore system of concrete or mortar, coupled with the migration of ions, concentration changes, and resulting phase changes, may alter its chloride penetration resistance and, subsequently, the time until rebar corrosion activation. Porosity changes in hardened Portland cement mortar were studied by means of mercury intrusion porosimetry (MIP) and electrochemical impedance spectroscopy (EIS), and alterations in the mortar surface phase composition were observed by means of X-ray diffraction (XRD). In order to innovatively investigate the impact of DC treatment on the properties of the mortar–electrolyte interface, the cathode-facing mortar surface and the anode-facing mortar surface were analyzed separately. The corrosion of steel coupons embedded in DC-treated hardened mortar was monitored by means of the free corrosion potential (E_oc_) and polarization resistance (R_p_). The results showed that the DC treatment affected the surface porosity of the hardened Portland cement mortar at the nanoscale. Up to two-thirds of the small pores (0.001–0.01 µm) were replaced by medium-sized pores (0.01–0.06 µm), which may be significant for chloride ingress. Although the porosity and phase composition alterations were confirmed using other techniques (EIS and XRD), corrosion tests revealed that they did not significantly affect the time until the corrosion activation of the steel coupons in the mortar.

## 1. Introduction

Direct current (DC)-based electrical treatments of concrete structures are used to improve the corrosion resistance of steel reinforcements embedded in concrete. One such treatment, cathodic protection, involves the use of an impressed direct current. Another treatment method involves electrochemical chloride extraction, where a DC is used to decrease the chloride content in the concrete cover layer between the surface of the concrete and the reinforcing bar. Also, the re-alkalization of carbonated concrete and electrochemical injections of inhibitors can be utilized to improve the microstructural integrity and corrosion resistance of reinforced concrete structures [1,2,3,4,5,6]. An electric current can be applied to obtain a rapid setting time for cement paste with a high-volume mineral admixture [7]. All such treatments are similar in principle and layout. In all cases, an external direct current source is required, which is connected by its negative pole to the reinforcement, thus serving as a cathode, and an external or built-in anode is connected to the positive pole. The direct current is then fed between the electrodes through the concrete. The charge between the electrodes is transferred via ion migration: positively charged cations (Ca^2+^, Na^+^, and K^+^) move toward the cathode, and negatively charged ions (Cl^−^ and OH^−^) move away from the cathode. DC treatments vary in terms of the duration of application, the current density flowing through the concrete, and the position of the anode and the surrounding environment. DC treatment can be used to restore favorable conditions on the steel surface, for example, through an electrochemical injection of migrating corrosion inhibitors [8,9].

In connection with the electrochemical treatment of concrete, changes in the properties of the cement binder can occur due to the passage of an electric current [10,11,12,13,14,15]. Paradoxically, the possible decomposition of the cementitious binder and the increased porosity could result in the deterioration of the concrete’s mechanical properties and increased transport of aggressive substances to the reinforcement. Regarding the mechanical properties, a reduction in the bond strength between the concrete and reinforcement and an induced alkali–aggregate reaction have been observed but at much higher current densities than those used in typical DC treatments [15]. Hydrogen embrittlement must be taken into account for high-strength steels [16]. A study focusing on the change in compressive strength concluded that the strength of carbonated concrete increased after re-alkalization treatment compared to that of noncarbonate concrete due to the precipitation of new solid material in the pores [5]. The larger pores were replaced by smaller ones, which was especially observable in the concrete near the cathode.

Changes in the cement binder due to electrochemical chloride extraction (ECE) were studied in detail by Thi [17]. Detailed analyses before and after DC treatment revealed that the calcium-to-silicon ratio in C-S-H gel changes significantly as a result of the concentration gradients of migrating ions in the pore solution induced by the current flow [15]. New phases with a very high calcium content are formed, and these later rearrange, probably to Portlandite. Such a change in the cement binder may affect the chloride binding properties and permeability, which is related to changes in porosity. Like mechanical properties, porosity changes strongly depend on the environmental conditions and the current density of the DC treatment. Susanto [15] found that no concentration gradient or limited convection leads to a decrease in porosity and the conversion of larger pores into smaller ones. If transport is not limited, for example, by immersion in water, then the effect is the opposite; that is, cumulative porosity increases, and small pores transform into larger ones, resulting in conditions favorable for increasing chloride penetrability.

There are many studies that address the effect of electrochemical treatments on the corrosion resistance of steel in concrete, but only as a consequence of changing the concentration of substances relevant to corrosion behavior. In particular, the chloride extraction rate [18], the degree of alkalinization of the pore solution around the reinforcement, or the introduction of a corrosion inhibitor to the reinforcement [19,20], as well as the two effects at the same time, as in the case of bidirectional electromigration rehabilitation [21], have been studied. The direct electrical current has also been considered as a tool for modifying the rheological properties and curing time of concrete [10,22,23].

In this paper, the aim was to study the effect of DC loading of hardened concrete or mortar with Portland cement binder in more detail than in previous publications. The current load was applied to mortar samples in order to investigate bulk and surface alterations in porosity and the cement binder’s chemical composition, as well as the subsequent impact of these alterations on the corrosion response of embedded steel coupons to penetrating chlorides. Mortar surface porosity and phase composition analysis and direct corrosion tests in DC-treated mortars have not been performed or reported before. Different current regimes were chosen to load the mortar with a wide range of passed charges. The loads were carried out in galvanostatic mode. The advantage of this approach is the maintenance of a constant charge flux and a clear definition of the total charge passed through the mortar samples of different thicknesses. If a constant voltage was used, the total charge and therefore the impact would be different for different thicknesses.

The evaluation of the effect of the passed charge consisted of an assessment of the key properties for chloride transport and, therefore, the concrete’s ability to protect against corrosion of the steel reinforcement. The change in porosity was evaluated by mercury intrusion porosimetry. Since chloride penetration through the cement binder can be significantly affected by structural and chemical changes on the mortar surface, the porosity of only the mortar surface was measured, separately for the surface facing the anode and separately for the surface facing the cathode during current loading of the mortar specimen. Both surfaces were also analyzed by X-ray diffraction. The change in the conductivity properties of the mortars was studied by high-frequency electrochemical impedance spectroscopy. Finally, the protective function of the mortars against chloride penetration was verified by measuring the free corrosion potential and polarization resistance on steel specimens embedded in current-loaded mortar.

## 2. Materials and Methods

### 2.1. Materials and Samples

Mortar samples were cast into cylindrical molds with a base diameter of 100 mm and a height of 200 mm. The samples were unmolded after 24 h and stored in saturated lime water for 90 days at ambient temperature (≈21 °C). After this curing period, the middle section of each cylinder was cut into three 10 mm disks for further testing by means of EIS, MIP, and XRD. Especially for EIS, the greater thickness would cause instability of AC signal and response recording due to the limited sensitivity of the electrochemical instrumentation at high mortar impedance. The mortar mixture consisted of 230.5 kg/m^3^ of CEM I 42.5 R Portland cement, 1289.0 kg/m^3^ of sand (0–4 mm), and 185.0 kg/m^3^ of water. The water-to-cement ratio was equal to 0.8. A relatively high water-to-binder ratio was selected for two reasons: to ensure the workability of the fresh mixture and to produce a lower-quality mortar, simulating damaged or distressed concrete typically targeted for repair. The initial total chloride content was lower than the detection limit of potentiometric titration of a hardened mortar powder leachate as prepared in accordance with the EN 14629 standard [24], which is app. 0.2 milligram of chlorides per 1 g of mortar.

**Figure 1:**
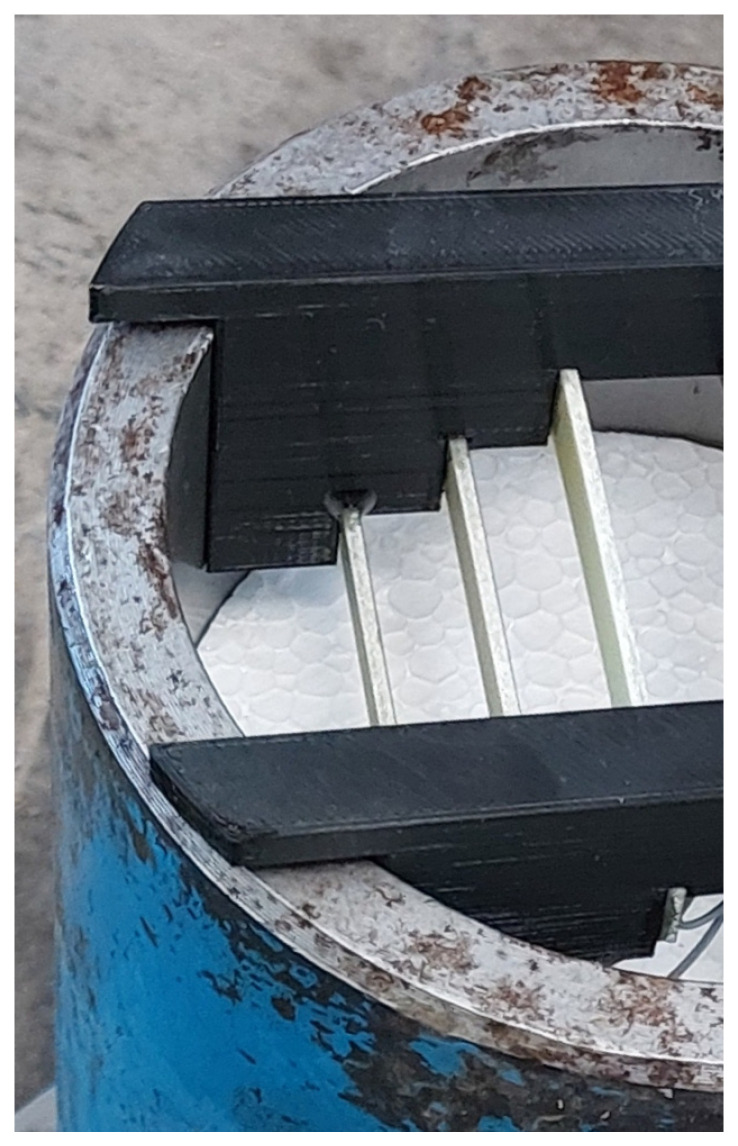
Position of corrosion coupons in a mold before casting.

The mortar samples with embedded steel corrosion coupons were produced and treated in the same manner, but no cutting was needed after casting and unmolding since the coupons were fixed inside the mold (Figure 1) using a specially designed holder, and the bottom of the mold was elevated so that the resulting height of the mortar sample with corrosion coupons was 50 mm.

**Figure 2:**
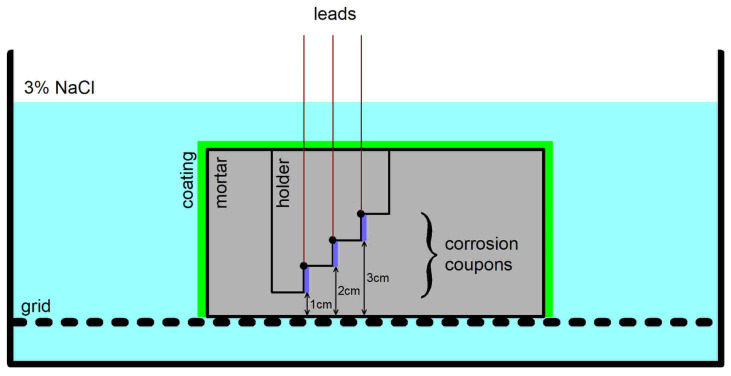
Schematic section of a mortar specimen with corroded steel coupons and its position during the diffusion test.

The corrosion coupons 10 × 70 mm were produced from unalloyed carbon steel sheet (EN 10130-1.0330 [25]) fixed on a polymeric substrate. A cable was attached to a corner of each coupon with a solder that was coated with two-component epoxy resin. The resulting surface area of the coupon was 6 cm^2^. Before embedding in mortar, all the coupons were cleaned with P600 emery paper and degreased with ethanol. The final position of the corrosion coupons in the mortar casting and the position of the mortar casting during the chloride diffusion test are shown in Figure 2.

### 2.2. Electrical Current Treatment

**Figure 3:**
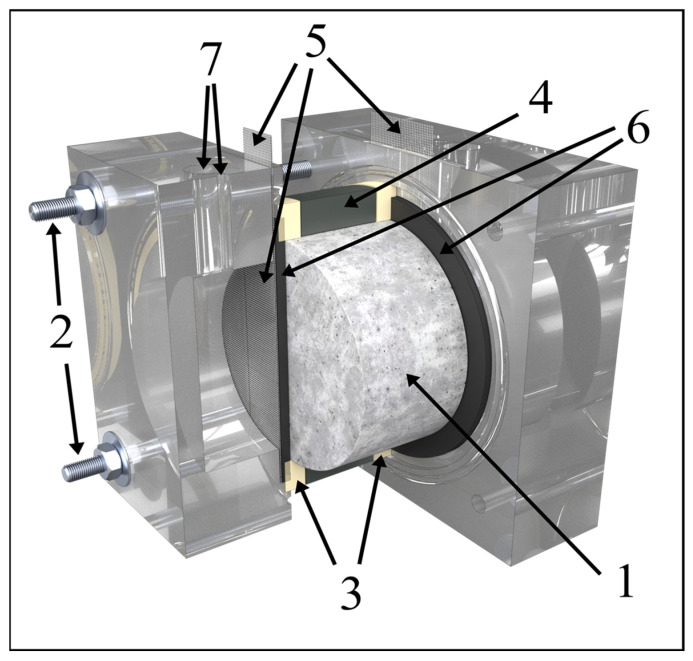
Scheme of the two-chamber cell: (1) sample, (2) retractable threaded rods, (3) rubber gasket, (4) central ring, (5) stainless-steel mesh electrodes, (6) graphite felt (for EIS only), and (7) pouring and venting openings.

Both the versions of the mortar samples (1 cm thick disks and 5 cm thick samples containing the corrosion coupons) were placed in a two-chamber cell between two AISI 316 mesh electrodes (Figure 3). Both chambers were filled with a saturated calcium hydroxide solution that was continuously exchanged with a fresh one during the treatment (approx. 2 L per day). A constant current density (1 A or 5 A per m^2^) for the mortar surface (79 cm^2^) was applied between the electrodes to achieve DC treatment for 1 day or 7 days. The total charge passed during DC treatment was 680 C for the 1 A/m^2^–1-day treatment, 4750 C for the 1 A/m^2^–7-day treatment, and 23,800 C for the 5 A/m^2^–7-day treatment. The corresponding charge density was 86 kC/m^2^, 601 kC/m^2^, and 3013 kC/m^2^, respectively.

The 1 cm thick samples were returned to a saturated calcium hydroxide storage solution after DC treatment. The 5 cm thick samples with the corrosion coupons were dried freely in a laboratory atmosphere overnight and then coated with a synthetic protective coating so that only one circular area of the mortar sample (the one facing the anode during the DC treatment) remained uncoated. After curing the two coating layers, the samples were also immersed in a saturated calcium hydroxide storage solution.

### 2.3. Electrochemical Impedance Spectroscopy

The arrangement seen in Figure 3 was used to collect the high-frequency electrochemical impedance spectra [26,27], with both chambers being filled with saturated calcium hydroxide solution. Impedance spectra were scanned with an electrochemical workstation Zahner Zennium X using the parameters provided in Table 1. The same arrangement applied for the DC treatment was used with constant voltage; the CeTech GFC030 graphite felt (3 mm thick) was only used to ensure a homogeneous distribution of the current over the mortar surface and to eliminate the electrolyte impedance. Electrochemical impedance spectra were collected 14 days after DC treatment. The samples were kept in saturated calcium hydroxide solution in the meantime. Working electrode leads of the potentiostat were always connected with the electrode attached to the anodic side of the mortar sample. The counter electrode lead, and the reference electrode lead was connected to the other electrode. ZView software (version 3.5f) was used for data evaluation using an equivalent circuit model. To verify the data using Kramers–Kronig transformation, Gamry EchemAnalyst 2 (version 7.10.4.14736) was employed.

### 2.4. Mercury Intrusion Porosimetry

Mercury intrusion porosimetry was performed in order to investigate the impact of DC treatment of concrete microstructure. Data were collected in the range of pore size from 0.0015 µm to 500 µm by means of AutoPore IV 9500.

Porosimetry was used to assess the change in the porosity of the mortar surface at the mortar-saturated calcium hydroxide solution interface due to current loading. Therefore, only the mortar surface that was in contact with the electrolyte during loading was subjected to mercury porosimetry. The other sides of the mortar samples were coated with two layers of Paraloid B72 applied as a 10% solution in xylene. Furthermore, the analysis distinguished whether the mortar surface faced the negative or positive electrode during loading. For this, we divided the loaded 10 mm thick mortar disk into two halves, one for the analysis of the positive side and the other for the analysis of the negative side. Each half was divided by fracture into approximately cubic pieces with a side size of approximately 1 cm. The fractured areas and the unanalyzed side were then coated with Paraloid. Ten such pieces were inserted into the analyzer each time.

### 2.5. X-Ray Diffraction

As was the case for mercury intrusion porosimetry, the surface of the mortar oriented to the negative electrode during loading and the surface oriented to the positive electrode were analyzed separately. A fragment of the mortar disk of approximately 2 × 2 cm was always selected for analysis. Measurements were performed at laboratory temperature on a θ–θ powder diffractometer, namely X’Pert PRO (Malvern Panalytical, Malvern, UK), in Bragg–Brentano para-focusing geometry using a CoKα radiation wavelength (λ = 1.7903 Å, U = 35 kV, I = 40 mA). Data were scanned using an ultrafast PIXcel^1D^ detector (Malvern Panalytical, Malvern, UK) in the 5–90° (2θ) angular range with a measurement step of 0.039° (2θ) and a reading time of 0.497 s/step. Data evaluation was performed with HighScore Plus 5.1 software and the PDF4+ reference sample database.

### 2.6. Corrosion Test

Corrosion coupons embedded in 50 mm thick mortar cylinders were used for the corrosion experiment (Figure 1). Mortar cylinders, both current-loaded and unloaded, were immersed in 3 wt.% NaCl solution, and the free corrosion potential (E_oc_) and polarization resistance (R_p_) were continuously monitored. The coupon potential was measured using a saturated calomel electrode (SCE), which was placed on the uncoated surface of the mortar cylinder for the measurement period only. The polarization resistance was evaluated from the linear polarization curve measured around the free corrosion potential from −20 mV (E_oc_) to +20 mV(E_oc_) at a polarization rate of 0.1 mV/s. The counter electrode made of AISI 316 stainless steel was placed in NaCl solution near the vessel wall, that is, as far away from the mortar cylinder as possible. The polarization resistance was evaluated as a slope of the measured dependence in the vicinity of ± 5 mV from the free corrosion potential recorded before polarization [28]. The value of the polarization resistance is taken as a measure of the corrosion rate, with the corrosion rate being directly proportional to the inverse of the polarization resistance. The Zahner Zennium X and Zahner Zennium E systems were used for the electrochemical measurements.

## 3. Results and Discussion

### 3.1. Conductivity Properties of Mortars

**Figure 4:**
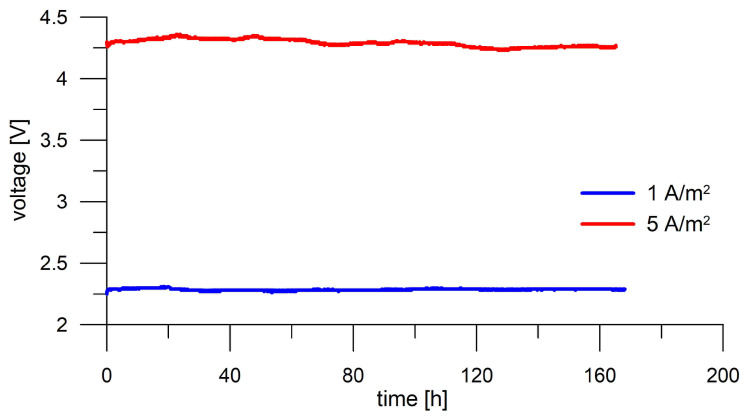
Voltage record during the seven-day current loading of 10 mm thick mortar disks.

**Figure 5:**
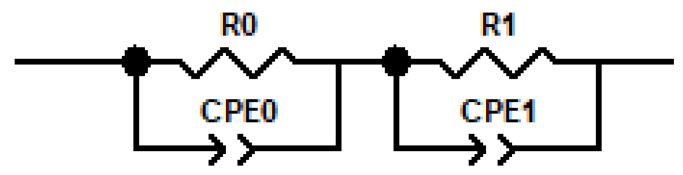
Equivalent circuit used for the evaluation of impedance spectra.

During current loads, the voltage between the load electrodes was recorded. It is obvious that, for higher current loads and for loading a mortar sample with a greater thickness, it was necessary to put more voltage on the electrodes. If this voltage varied during loading, it would probably be related to a change in the microstructure of the cement binder or the continuous pores in it, which are crucial for ion transport [29]. It is clear from the voltage recording examples during loading of 10 mm thick mortars that no significant voltage changes occur (Figure 4). High-frequency electrochemical impedance spectroscopy, expressed in terms of admittance data, was employed to evaluate changes in mortar conductivity induced by DC treatment. For data evaluation, the conventional equivalent circuit model for concrete proposed by Song et al. (Figure 5) was employed [30], which provides a balance between accurate fitting of the impedance spectra and direct interpretability of the extracted parameters in terms of the microstructural features of the mortar, with capacitors replaced by constant phase elements (CPE) defined as Z = (1/Y_0_)(jω)^α^ to account for the non-ideal distribution of impedance in such a heterogeneous material [30]. The real capacitance of CPE was determined using ω_max_ in accordance with the method of Hsu and Mansfeld [31]. The characteristic parameters—including the resistance or the conductivity of the continuously conductive path (R_CCP_/G_CCP_), the resistance or conductivity of discontinuous pores (R_cp_/G_cp_), the interfacial capacitance in the discontinuous path (C_DP_), and the capacitance of the non-conductive matrix (C_mat_)—were calculated based on equations defined within the equivalent circuit model [30]. Impedance spectra were measured on 10 mm thick samples saturated with lime water before loading and two weeks after loading. During these two weeks, the samples also relaxed in lime water.

**Figure 6:**
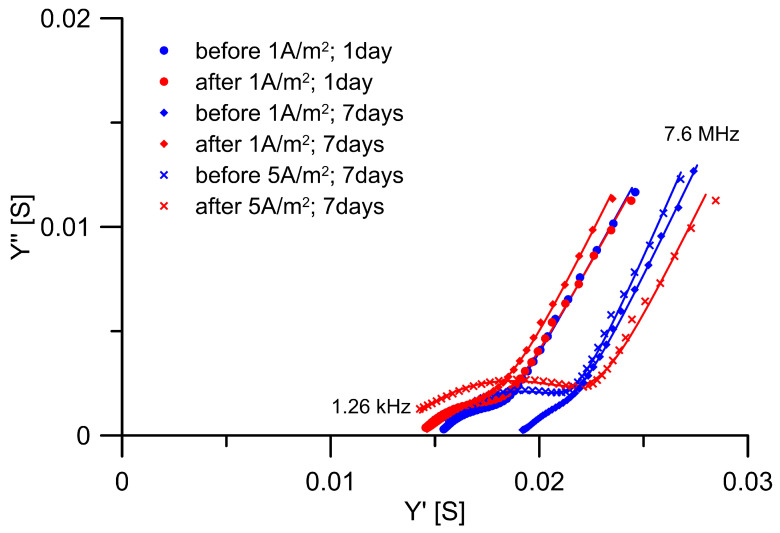
Nyquist representation of the admittance spectra for the mortar disk (blue—before load; red—after load; the red and blue solid lines represent the fits of experimental); Y′ and Y″ represent the real and imaginary components of the admittance.

Figure 6 presents the admittance data of the mortar samples before and after DC treatment. In all spectra, two characteristic time constants are present, consistent with theoretical expectations [30]. These are represented in the model by the R_0_—CPE_0_ and R_1_—CPE_1_ pairs, from which the characteristic parameters listed in Table 2 were calculated.

All samples exhibit consistent trends: only minimal changes (less than 10%) in the capacitance of the material matrix (C_mat_); a decrease (up to 26%) in the conductivity of continuous pathways (G_CCP_); and a pronounced reduction (up to 33%) in pore resistance (R_CP_), accompanied by an increase (up to 49%) in interfacial capacitance along the discontinuous conductive path (C_DP_). All these changes become more pronounced with longer DC exposure duration. As predicted by Susanto et al., the increase in current during DC treatment does not lead to an increase in the conductivity of the continuous conductive path; however, the results reveal a strong time-dependent effect under identical loading conditions (1 A/m^2^): while only a modest 6% relative change of G_CCP_ was observed after 1 day of treatment, a marked 26% decrease became evident after 7 days [15].

### 3.2. Porosity of Mortars

**Figure 7:**
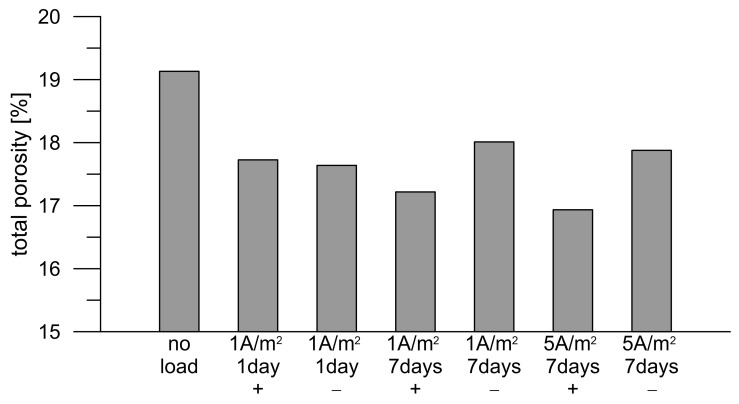
Total porosity of Portland cement mortar before and after various current loads (+ and − refer to polarity of the electrode adhering to the surface of the mortar disk).

The higher electrical resistance after current loading should be reflected in the porosity and penetrability of the chlorides through the mortar. Reduced penetrability has been confirmed by diffusion tests on concrete cylinders, the results of which are published elsewhere [32]. The MIP confirmed that total porosity decreased approximately by 10% due to the DC treatment compared to the total porosity of the current-unloaded mortar, while it was not significant for the total porosity value whether the analyzed mortar surface faced to the anode or the cathode during load (Figure 7). However, such a decrease in porosity could be significant for the overall protective function of the mortar or concrete with respect to corrosion of the embedded reinforcement. Therefore, the mortar was subjected to a more detailed analysis.

**Figure 8:**
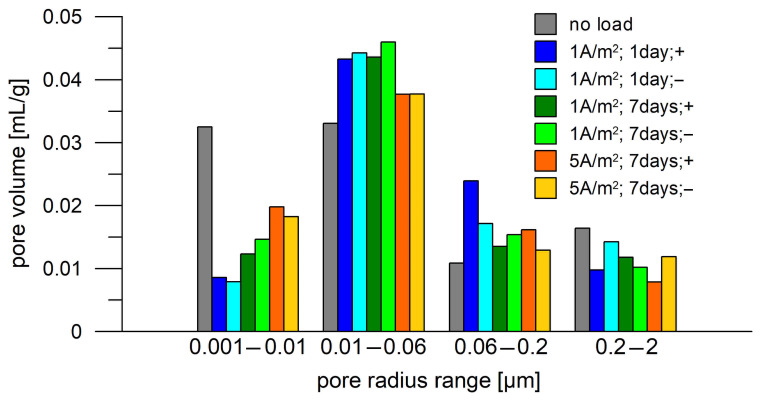
Distribution of porosity of Portland mortar before and after various current loads, distinguishing between its surface facing cathode (−) or anode (+).

Changes in porosity can be seen in more detail when comparing porosity with pore size (Figure 8). The pore size distribution of the mortars after different DC treatments resulting from the MIP was separated into four pore radius intervals (Figures S1 and S2). The selected ranges correspond to pore radius intervals in which the pore volume change related to the pore radius (-dV/d log r) of DC-treated mortar differs significantly from that of untreated mortar. Typically, the limits of the intervals correspond to the pore radius value in which the difference is minimal or low. In Figures S1 and S2, the ranges have been highlighted with color fields. For the Portland cement mortar, the current load causes a decrease in the volume of small pores (0.001–0.01 µm), which are probably replaced by larger pores (0.01–0.06 µm). This is most pronounced at low current loads (1 A/1 day). Approximately 2/3 of small pores (0.001–0.01 µm) were replaced by medium-sized pores (0.01–0.06 µm and 0.06–0.2 µm) as a result of DC treatment with 1 A/m^2^. At longer and higher loads, the decrease in the volume of small pores and the increase in the volume of larger pores are smaller (40–60%). The volume of the larger pores (>0.06 µm) changes in a nonsystematic manner; however, overall, an increase in the pore volume of the 0.06 to 0.2 µm interval and a decrease in the pore volume of the 0.2 to 2 µm interval after current loading are more likely to be observed, with the difference from the unloaded sample decreasing with increasing passed charge. This suggests that significant changes in the microstructure of the cement binder occur in the initial phase of current loading, and as the electric charge continues to pass, new microstructural changes occur that eliminate the initial change. No difference was observed between the porosity of the side facing the negative and positive electrodes. From the point of view of the penetrability of aggressive ions into concrete, this could be a positive finding, as the overall impact of a long-term electric current flow on chloride penetration might not be significant.

### 3.3. X-Ray Diffraction Analysis of the Mortar Surface

Changes in porosity on the surface of current-loaded mortars could be related to ions’ local concentration changes, induced by their migration. The disturbance of the chemical equilibrium or at least of the steady state between the solid phases of cement mortar and the surrounding solution may induce, on the one hand, the dissolution of some solid phases and, on the other hand, the precipitation of secondary phases with lower solubility under newly established conditions [17].

Both surfaces of the current-load mortar were also analyzed by X-ray diffraction (Table 3). The individual XRD patterns and protocols are provided in the Supplementary Materials (Protocols S1–S7). The dominant phases identified are, as expected, quartz and aluminosilicates (identified as Albite and Microcline), with calcite, a product of carbonation of the calcareous components, also being found. Although the XRD results were evaluated only semiquantitatively, it is clear that quartz dominates over the calcareous phase in most of the surface samples, and the difference between the current-impacted samples and the unloaded sample is not significant. The difference in the ratio of silicate to calcium phases between the surfaces of the loaded samples adjacent to the positive and negative electrode appears to be significant. At the cathode, the proportion of the carbonate phase, i.e., calcite, increases relative to quartz. A possible explanation for this is the effect of the passing electric charge, which causes all ions, including calcium, to migrate, resulting in concentration changes at the solution–solid phase interface. The accumulation of calcium ions together with the increase in pH at the cathode leads to the solubility limit of the Portlandite being reached and its precipitation at the phase interface. The source of these calcium ions is the Portlandite deposited in the mortar pore system and C-S-H gel, both of which are thereby disturbed, and the microstructure of the mortar is then affected, resulting in the porosity changes described above.

The question remains as to why the XRD did not identify the remaining calcareous phase as Portlandite, i.e., the noncarbonated phase. It is likely that the surface of the analyzed sample had succumbed to carbonation in the period prior to analysis, although both the sample shortly after the end of the current load and the sample without the current load were thoroughly rinsed with water and placed in acetone to remove water after removal from the preservation solution.

### 3.4. Corrosion Test in Mortar

Alteration of a cement binder’s microstructure via the passage of a DC electric current has been demonstrated not only by this study but also by several other authors [13,14,15,17,22]. The key question for the practical applications for real reinforced concrete structures is whether this influence is significant for the penetrability for chloride anions and the subsequent depassivation of steel reinforcement embedded in concrete. For this reason, corrosion tests were performed in this study to investigate the corrosion response of steel specimens embedded in Portland cement mortar, which were subjected to DC treatment after curing and subsequently subjected to chloride diffusion testing.

**Figure 9:**
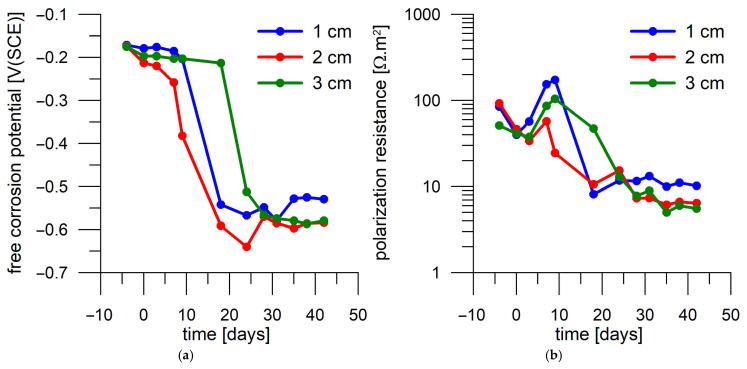
Corrosion monitoring of steel coupons in the first mortar cylinder loaded with 1 A/m^2^ for one day during immersion in a 3 wt.% NaCl solution. Day 0 corresponds to the start of exposure in 3 wt.% NaCl solution. (**a**) Free corrosion potential record; (**b**) polarization resistance record.

**Figure 10:**
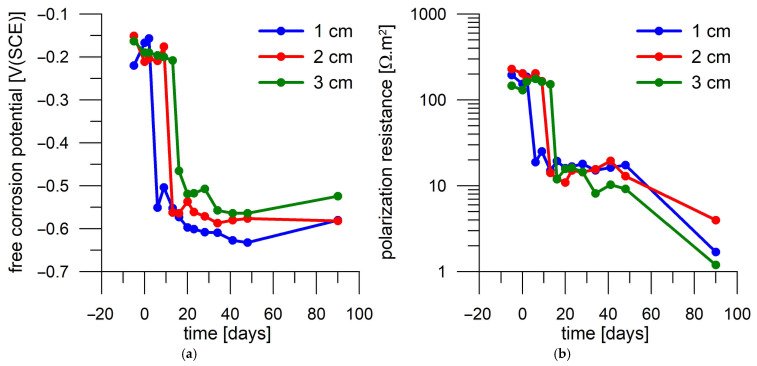
Corrosion monitoring of steel coupons in the second mortar cylinder loaded with 1 A/m^2^ for one day during immersion in a 3 wt.% NaCl solution. Day 0 corresponds to the start of exposure in 3 wt.% NaCl solution. (**a**) Free corrosion potential record; (**b**) polarization resistance record.

**Figure 11:**
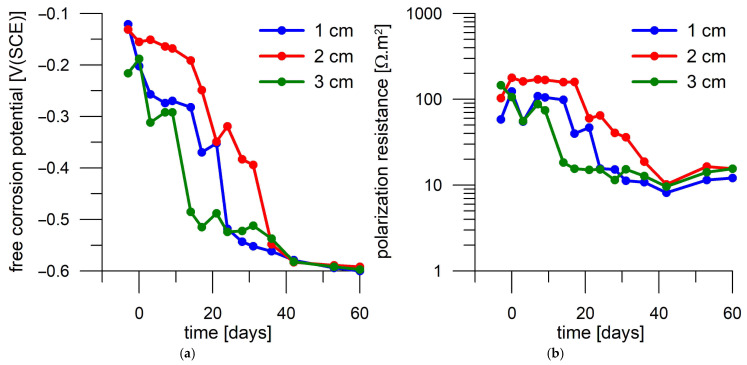
Corrosion monitoring of steel coupons in the first mortar cylinder without current loading during immersion in 3 wt.% NaCl solution. Day 0 corresponds to the start of exposure in 3 wt.% NaCl solution. (**a**) Free corrosion potential record; (**b**) polarization resistance record.

**Figure 12:**
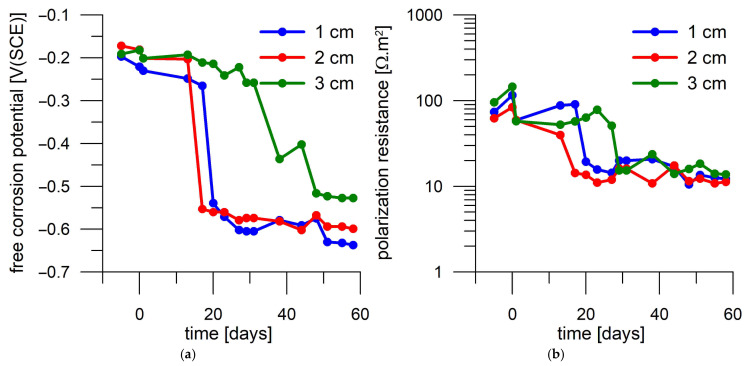
Corrosion monitoring of steel coupons in the second mortar cylinder without current loading during immersion in 3 wt.% NaCl solution. Day 0 corresponds to the start of exposure in 3 wt.% NaCl solution. (**a**) Free corrosion potential record; (**b**) polarization resistance record.

**Figure 13:**
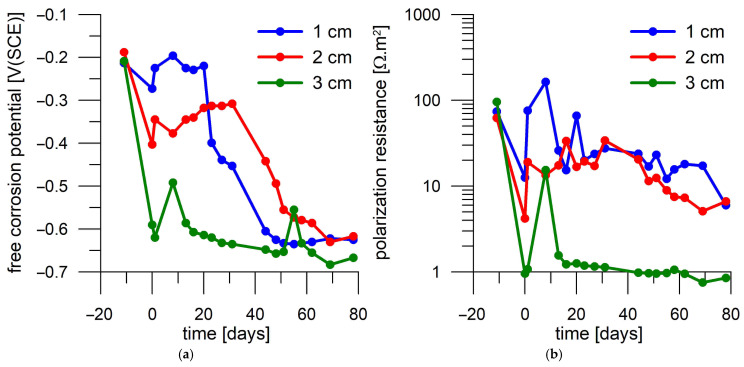
Corrosion monitoring of steel coupons in mortar cylinders loaded with 5 A/m^2^ for seven days during immersion in a 3 wt.% NaCl solution. Day 0 corresponds to the start of exposure in 3 wt.% NaCl solution. (**a**) Free corrosion potential record; (**b**) polarization resistance record.

Firstly, the corrosion responses of steel samples were compared in mortars without current load and with 1 A/m^2^ load for 1 day. Three identical mortar cylinders, each with three embedded steel samples at three different distances from the mortar surface through which chloride from the surrounding 3 wt.% NaCl solution passed, were used for this comparison. There were two replicates of the mortar samples subjected to the same DC treatment, as presented in separate figures (Figure 9 and Figure 10 for 1 A/m^2^—1 day and Figure 11 and Figure 12 for the non-DC-treated samples). One of the mortar cylinders was also subjected to a current load of 5 A/m^2^ for 7 days (Figure 13) prior to the diffusion test to assess the long-term effect of the current load. Figure 9, Figure 10, Figure 11, Figure 12 and Figure 13 plot the records of free corrosion potentials and polarization resistances of each coupon during the corrosion test. A time of 0 days on the x-axis corresponds to the day on which the mortar cylinder was placed in a 3 wt.% NaCl solution and the diffusion of chloride into the mortar was initiated. Therefore, for example, for one of the samples loaded with a current density of 1 A/m^2^ for 1 day (Figure 9), a value of the free corrosion potential and polarization resistance was measured 3 days before immersion in 3 wt.% NaCl solution, then on the day of immersion, and then after 3 days of exposure in 3 wt.% NaCl solution. There is a nonlinear decrease in both values during exposure. Two regions in which the values are relatively stable can be distinguished. In the case of E_oc_, there is a region of values more positive than −300 mV(SCE) and values more negative than −500 mV(SCE), and in the case of R_p_, there are a region of values higher than 30 Ω.m^2^ and a region of R_p_ lower than this value. It is known from the literature [33] that this value is considered to be the limit for the acceptable and unacceptable corrosion rate of steel reinforcement in concrete.

In terms of E_oc_, it is likely that the region of values more positive than −300 mV corresponds to a condition where the steel sample remains in a passive state, and thus the chlorides probably have not yet reached the depth of sample position in the quantities required to break the passive layer. On day 18 of exposure in 3 wt.% NaCl solution, the E_oc_ of the two samples closest to the surface was already more negative than −500 mV(SCE); therefore, it can be assumed that the passive layer broke and started transitioning to the active state (Figure 9a). The deepest coupon remained passive after 18 days of exposure. Its activation occurred within 24 days. This corresponds to polarization resistance that changed the values from the region of an acceptable corrosion rate (Rp > 30 Ω.m^2^) to the region of an unacceptable corrosion rate on the same days (Figure 9b).

Thus, it seems that by monitoring E_oc_ and R_p_, it would be possible to follow the progress of the chloride front through the mortar and use the time to activation to assess the effect of current loading on chloride penetrability. However, this comparison appears difficult when considering the results provided by another DC-treated mortar sample in an identical manner. In that case, a decrease in E_oc_ is observed on day 6 after immersion in a 3 wt.% NaCl solution (Figure 10a) for the steel coupon located closest to the mortar surface. Another steel coupon was activated on day 13, namely the sample at a medium distance from the surface. The deepest sample was not activated until three days later. Therefore, in the case of the second mortar sample, the activation of the embedded steel samples occurred earlier, even though both evaluated mortar samples were produced and processed in the same way.

In summary, taking both the DC-treated (1 A/m^2^ for 1 day) mortar samples into account, the time to activation of a coupon located 1 cm from the surface was 18 to 21 days, while that of a coupon at 2 cm from the surface was 13 to 18 days, and the coupon at 3 cm was activated between day 16 and 24 (Table 4).

**Figure 14:**
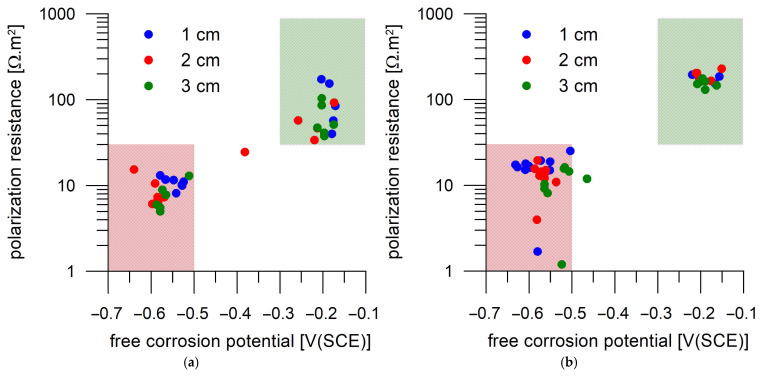
Relation of the free corrosion potential and the steel polarization resistance of steel in terms of passivity (green background) and activity (red background) for two identical Portland cement mortar samples (**a**,**b**) loaded with 1 A/m^2^ for one day.

The qualitative distribution of the corrosion behavior of the steel specimens to the passive active state can be seen in Figure 14, in which the individual points are divided into two groups, with the passive state corresponding to the group in the upper right green field and the active state corresponding to the group in the lower left red field. Such a distribution agrees well with the data of Alonso et al. [34], according to whom the active corrosion is considered when the corrosion rate of the rebar is higher than 0.1 μA/cm^2^. According to the RILEM TC 154-EMC [35], the recommended value is B = 26 mV. This means that active corrosion can be expected if the R_p_ value is below 26 Ω.m^2^. Since the evaluation of the resistance is always subject to a certain error, it is justified to take this limit value with a bit of consideration. Taking into account the E_oc_ values, which are clearly separated into two groups, Figure 14 confirms that a value of 30 Ω.m^2^ as a threshold between the active and passive corrosion is reasonable.

Steel coupons deposited in unloaded mortar also exhibit passive behavior for several days before a decrease in E_oc_ and R_p_ is observed. However, there are cases where the steel coupon embedded deeper in the mortar is activated before the coupon closer to the surface. For example, the coupon deposited at 3 cm shows active behavior after 14 days according to both E_oc_ and R_p_, while the coupon at 1 cm depth shows active behavior after 24 days (Figure 11). The middle sample shows a gradual decrease in E_oc_ and R_p_, and thus, it is not possible to accurately determine the time to activation for this coupon.

The activation of the first two steel coupons deposited in the other unloaded mortar cylinder is evident due to the drop in E_oc_ and R_p_ after 17 and 20 days, respectively. The time to activation of the deepest deposited sample is not clear. According to R_p_, activation occurs on day 29, and according to E_oc_, it occurs only after 48 days (Figure 12).

Table 4 summarizes the times to activation of each steel coupon as derived from E_oc_ and R_p_ and provides an evaluation of the effect of current loading on the time to activation of corrosion specimens embedded in mortar, as demonstrated by corrosion tests. If it is assumed that the time to activation is determined by reaching the critical chloride concentration for passive layer breakdown at a given depth [36], then it can be concluded that the chosen current load does not have a significant effect on the chloride penetrability of the mortar. Evaluations of the impact of DC treatment on chloride diffusion have been published earlier [32,37], but in the case of corrosion testing, other more significant heterogeneities in the mortar pore system, like preferential local pathways for chloride penetration to corrosion coupons, are likely to be involved in the activation. Therefore, the activation of corrosion coupons occurs earlier than would be expected from the progression of the chloride front, which is the result of analysis of a homogenized layer taken over a certain depth of the sample and thus does not capture locally deeper chloride penetrations.

To confirm the insignificant effect of current loading on the time to steel activation in mortar, a significantly higher current load of mortar with embedded corrosion coupons was performed. For 7 days, the mortar sample was loaded with a current density of 5 A/m^2^. However, it appeared that the current load affected the corrosion behavior of the coupons even before diffusion saturation of the affected mortar with chlorides, and the activation of some coupons occurred even without the presence of chlorides. The three corrosion coupons exhibited high E_oc_ (approximately −200 mV(SCE)) and R_p_ (app. 70–100 Ω.m^2^) values before current loading. However, after the end of the load, all samples showed a loss of passivity with a decrease in R_p_ to 10 or even 1 Ω.m^2^, as well as a decrease in E_oc_ to −600 mV(SCE). The most pronounced decrease in values was observed for the sample deposited deepest, while the lowest decrease was observed for the sample closest to the surface, only showing a slight decrease in E_oc_ (Figure 12). The corrosion activation was probably induced by the formation of an electrolytic cell within the corrosion coupons, which were located in a relatively strong voltage field induced in the mortar cylinder. This activation is not stable, and the corrosion samples tend to recover their passivity in the non-aggressive mortar after the current load is terminated, as manifested by an increase in both E_oc_ and R_p_ in the first 10 days of immersion in NaCl solution (Figure 12). This is then followed by a decrease in both values again in the following days, probably because chloride reached the surface of the corroded samples. For the deepest sample and for the sample at the lowest depth, this probably occurred after 13 days. For the middle sample, reactivation is not evident because there was no clear recovery of the high R_p_ values. However, from the decreasing trend of E_oc_, it can be inferred that activation occurred after day 30 of the diffusion test.

## 4. Conclusions

The impact of the DC treatment of Portland cement mortar on its surface porosity and mineral phase composition was studied using MIP, EIS, and XRD. The impact of DC treatment on the properties of the mortar–electrolyte interface, the cathode facing the mortar surface, and the anode facing mortar surface were analyzed separately in an innovative manner. We also evaluated the significance of the alteration in the surface properties and their effect on subsequent steel corrosion behavior in the mortar. The response of steel coupons embedded in the mortar at various depths was monitored by means of free corrosion potential and polarization resistance. The following conclusions were drawn:

- The conductivity properties of Portland cement mortar were influenced by DC treatment. The largest decrease (26%) in the conductivity of the mortar’s continuous pathways was observed for the less intensive DC treatment, 1 A/m^2^ applied for 7 days. Under more intensive DC treatments, a lower decrease in conductivity was identified.
- DC treatment alters the porosity of mortar surface layers, especially affecting the distribution of pore volume in relation to pore size. Approximately 2/3 of small pores (0.001–0.01 µm) were replaced by medium-sized pores (0.01–0.06 µm) as a result of 1 A/m^2^ DC treatment. The porosity alteration was not that pronounced for the higher-intensity DC treatment, a finding that is in agreement with the above-mentioned changes in conductivity properties.
- Alteration of the mortar surface was also confirmed by a phase analysis revealing an increased carbonate phase content, likely attributable to the carbonation of Portlandite precipitated in catholyte.
- Corrosion monitoring of the steel coupons embedded in Portland cement mortar showed a clear relation between the free corrosion potential (E_oc_) and the resistance to polarization (R_p_). In most cases, the passive state was characterized by R_p_ > 30 Ω.m^2^ and E_oc_ > −300 mV(SCE), while the active state was characterized by R_p_ < 30 Ω.m^2^ and E_oc_ < −500 mV(SCE).
- The transition of the corrosion coupons embedded in the Portland cement mortar from the passive to the active corrosion state occurred as early as after 13 days of immersion in 3 wt.% NaCl solution or later (up to 36 days) regardless of the coupons’ distance from the mortar surface (1–3 cm) and the DC treatment.

Overall, it can be concluded that the changes in mortar properties due to the DC passage observed via MIP, EIS, and XRD are not essential for the corrosion behavior of steel embedded in Portland mortar. Since the DC treatment in this study was applied for a limited time (up to 7 days), this conclusion could be revised if the mortar was treated for a longer period or continuously. This could provide motivation for future studies in which mortars would be loaded for a longer period or with higher DC values. However, as observed in this study, high current loads would affect the corrosion of the reinforcement more directly by the formation of an electrolytic cell on the reinforcement than by changing the microstructure of the cementitious matrix. A more significant factor in the time to activation, at least in the chosen corrosion experiment setup, will likely be the preferential pathways for local chloride transport other than those observed by MIP and EIS. Taking into account all the obtained results, especially the corrosion behavior of steel coupons in mortar affected by direct current treatment, it can be concluded that a DC passage of the magnitude commonly used for remediation treatment of reinforced concrete structures, e.g., electrochemical chloride extraction, cathodic protection, or re-alkalization, does not adversely affect the transport properties of the mortar to such an extent that the time to activation of the steel embedded within it is shortened.

## Figures and Tables

**Table 1:** Parameters of electrochemical impedance spectroscopy.

Lower frequency limit	1.26 Hz
Upper frequency limit	7.6 MHz
Steps per decade	10
Measure periods	50
Frequency scan direction	Up–down
Frequency scan strategy	Single sine
Amplitude	20 mV

**Table 2:** Impedance of mortar disks (10 mm thick) before and after current load and its relative change.

Current Load	1 A/m^2^; 1 Day		1 A/m^2^; 7 Days		5 A/m^2^; 7 Days	
R_cp_ [Ω]	349	261	338	227	154	103
R_ccp_ [Ω]	65	70	53	70	66	75
C_dp_ [F]	1.3 × 10^−8^	1.6 × 10^−8^	4.6 × 10^−9^	6.3 × 10^−9^	3.6 × 10^−8^	9.1 × 10^−8^
C_mat_ [F]	2.4 × 10^−10^	2.3 × 10^−10^	2.5 × 10^−10^	2.3 × 10^−10^	2.4 × 10^−10^	2.2 × 10^−10^
G_cp_ [S]	2.9 × 10^−3^	3.8 × 10^−3^	3.0 × 10^−3^	4.4 × 10^−3^	6.5 × 10^−3^	9.7 × 10^−3^
G_ccp_ [S]	1.5 × 10^−2^	1.4 × 10^−2^	1.9 × 10^−2^	1.4 × 10^−2^	1.5 × 10^−2^	1.3 × 10^−2^

**Table 3:** Semiquantitative XRD analysis (weight %) of mortar disk surface before and after various current loadings (+ and − refer to the polarity of electrode adhering to the surface of the mortar disk).

Current Load	Quartz	Aluminate–Silicate Phases	Carbonate Phases	Rest	Supplementary Material
No load	85	5	5	5	Protocol S1
1 A/m^2^; 1 day; +	80	8	6	6	Protocol S2
1 A/m^2^; 1 day; −	75	10	14	1	Protocol S3
1 A/m^2^; 7 days; +	80	12	5	3	Protocol S4
1 A/m^2^; 7 days; −	50	9	40	1	Protocol S5
5 A/m^2^; 7 days; +	70	17	10	3	Protocol S6
5 A/m^2^; 7 days; −	55	10	35	-	Protocol S7

**Table 4:** Time to activation of corrosion coupons embedded in Portland mortar samples after immersion in 3 wt.% NaCl solution.

Time to Activation [Days]	Depth 1 cm	Depth 2 cm	Depth 3 cm
No load	20–24	17–36	14–29
1 A/m^2^; 1 day	18–21	13–18	16–24
5 A/m^2^; 7 days	13–21	17–31	13

## Data Availability

The raw data supporting the conclusions of this article will be made available by the authors on request.

## Supplementary Materials

The following supporting information can be downloaded at: https://www.mdpi.com/article/10.3390/ma18143365/s1, Figure S1: Distribution of porosity of Portland cement mortar before and after various current loads—mortar surface facing anode (+); Figure S2: Distribution of porosity of Portland cement mortar before and after various current loads—mortar surface facing cathode (−); Protocols S1–S7: XRD patterns and protocols for both surfaces of the Portland cement mortar before and after various current loads.

## Abbreviations

The following abbreviations are used in this manuscript:

DC	Direct current
MIP	Mercury intrusion porosimetry
EIS	Electrochemical impedance spectroscopy
XRD	X-ray diffraction
E_oc_	Free corrosion potential or open-circuit potential
R_p_	Polarization resistance
ECE	Electrochemical chloride extraction
C-S-H	Calcium hydroxyzincate
SCE	Saturated calomel electrode
R	Resistance
CPE	Constant phase element
R_CCP_	Resistance of the continuously conductive path
G_CCP_	Conductivity of the continuously conductive path
R_cp_	Resistance of discontinuous pores
G_cp_	Conductivity of discontinuous pores
C_DP_	Interfacial capacitance in the discontinuous path
C_mat_	Capacitance of the non-conductive matrix
