# Supplementary material for: Corrosion Response of Steel to Penetration of Chlorides in DC-Treated Hardened Portland Cement Mortar
Source: Materials (Basel). 2025 Jul 17;18(14):3365. doi: 10.3390/ma18143365 (PMC12300289; doi:10.3390/ma18143365)
Supplement: Supplementary file 1 [file materials-18-03365-s001.zip › protocol s1.pdf]

Protocol S1

Measurement Conditions:

Dataset Name	XADS20_5-90_T120_s6_sp0_rp0_MA-27
File name	\\share\rentgenka980\PC2_XPERT\2024\Kouril\2024-04-23\XADS20_5-90_T120_s6_sp0_rp0_MA-27.xrdml
Sample Identification	MA-27
17min50s	
PHD Lower Level = 4.02 (keV), PHD Upper Level = 9.70 (keV)	
Measurement Start Date/Time	02.05.2024 15:55:00
Operator	localadmin
Raw Data Origin	XRD measurement (*.XRDML)
Scan Axis	Gonio
Start Position [°2θ]	4,8647
End Position [°2θ]	89,8067
Step Size [°2θ]	0,0390
Scan Step Time [s]	116,5350
Scan Type	Continuous
PSD Mode	Scanning
PSD Length [°2θ]	3,35
Offset [°2θ]	0,0000
Divergence Slit Type	Fixed
Divergence Slit Size [°]	1,0000
Specimen Length [mm]	20,00
Measurement Temperature [°C]	25,00
Anode Material	Co
Intended Wavelength Type	K-α1
K-α1 [Å]	1,78901
K-α2 [Å]	1,79290
K-β1 [Å]	1,62083
K-β2 [Å]	1,38113
K-β3 [Å]	1,39261
K-A2 / K-A1 Ratio	0,50000
K-Alpha2 Line Shift	0,00000
K Absorption Edge	1,37868
Generator Settings	40 mA, 35 kV
Diffractometer Type	0000000080910230
Diffractometer Number	0
Goniometer Radius [mm]	240,00
Dist. Focus-Diverg. Slit [mm]	100,00
Incident Beam Monochromator	No
Spinning	No
Fast detector	PIXcel1D_1D detector

Main Graphics, Analyze View:

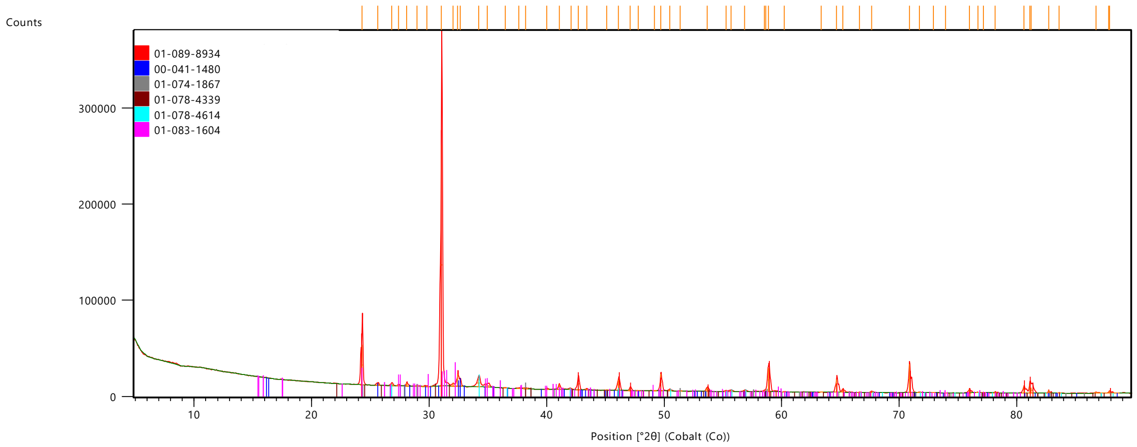

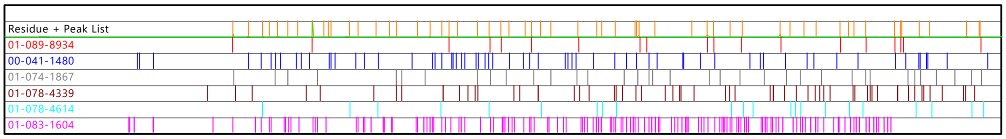

Peak List:

Pos. [°2θ]	d-spacing [Å]	Height [cts]	Rel. Int. [%]	FWHM Left [°2θ]	Matched by
24,2691	4,25533	41739,18	16,63	0,1913	01-089-8934, 01-074-1867, 01-083-1604
25,6191	4,03456	2602,56	1,04	0,1199	00-041-1480
26,8165	3,85749	1949,02	0,78	0,1985	00-041-1480, 01-078-4614, 01-083-1604
27,3997	3,77690	1546,24	0,62	0,0971	00-041-1480, 01-083-1604
28,0846	3,68658	2922,89	1,16	0,1405	00-041-1480, 01-074-1867
28,9808	3,57491	500,11	0,20	0,0780	01-074-1867, 01-083-1604
29,7967	3,47914	376,08	0,15	1,0088	00-041-1480, 01-083-1604
31,0411	3,34289	251018,40	100,00	0,1411	01-089-8934, 01-083-1604
32,0526	3,24004	2899,33	1,16	0,0900	01-083-1604
32,4335	3,20299	12314,40	4,91	0,1109	00-041-1480
32,6311	3,18412	4670,89	1,86	0,1007	00-041-1480
34,2222	3,04020	8072,19	3,22	0,2708	01-078-4614
34,9303	2,98043	2456,02	0,98	0,4664	00-041-1480, 01-083-1604
36,4924	2,85692	701,43	0,28	0,0920	00-041-1480,

					01-078-4339, 01-078-4614
37,6265	2,77379	278,14	0,11	0,3806	01-083-1604
38,2111	2,73290	679,43	0,27	0,4644	01-074-1867, 01-078-4339
39,9967	2,61556	933,38	0,37	0,1716	01-083-1604
41,0707	2,55001	4887,36	1,95	0,1255	01-083-1604
42,0861	2,49117	519,90	0,21	0,4147	00-041-1480, 01-078-4339, 01-078-4614, 01-083-1604
42,6858	2,45777	11718,59	4,67	0,1449	01-089-8934, 01-083-1604
43,4148	2,41845	1134,87	0,45	0,1608	00-041-1480, 01-078-4339
45,1014	2,33247	439,25	0,17	0,0780	00-041-1480, 01-074-1867, 01-078-4339
46,1029	2,28448	10647,66	4,24	0,1752	01-089-8934, 01-074-1867, 01-078-4614
47,1208	2,23785	3252,30	1,30	0,0911	01-089-8934
47,8125	2,20734	164,44	0,07	0,0780	00-041-1480, 01-083-1604
49,1722	2,14994	96,54	0,04	0,2306	00-041-1480, 01-083-1604
49,7240	2,12758	17360,23	6,92	0,1223	01-089-8934, 00-041-1480, 01-074-1867, 01-083-1604
50,4758	2,09792	1784,37	0,71	0,1177	00-041-1480, 01-078-4339, 01-078-4614
51,3732	2,06369	521,39	0,21	0,0780	01-074-1867, 01-083-1604
53,6567	1,98198	3952,36	1,57	0,2238	01-089-8934, 00-041-1480, 01-074-1867, 01-078-4339
55,2785	1,92821	800,91	0,32	0,3947	01-078-4614
55,6913	1,91506	1231,34	0,49	0,2556	01-078-4614, 01-083-1604
56,8616	1,87883	1293,40	0,52	0,2973	00-041-1480, 01-078-4339, 01-078-4614, 01-083-1604
58,5445	1,82940	873,49	0,35	0,1550	00-041-1480, 01-074-1867, 01-078-4339, 01-083-1604

58,6590	1,82615	1488,74	0,59	0,1284	00-041-1480, 01-074-1867, 01-078-4339, 01-083-1604
58,8753	1,82004	24121,66	9,61	0,1915	01-089-8934, 01-074-1867, 01-078-4339, 01-083-1604
60,2206	1,78307	483,56	0,19	0,0780	00-041-1480, 01-074-1867, 01-083-1604
63,3536	1,70341	0,00	0,00	0,0780	01-078-4339
64,6661	1,67247	13142,49	5,24	0,1831	01-089-8934, 01-074-1867, 01-083-1604
65,1986	1,66030	3490,40	1,39	0,1126	01-089-8934, 00-041-1480, 01-083-1604
66,6230	1,62877	93,31	0,04	0,0780	01-078-4614, 01-083-1604
67,6375	1,60718	1125,61	0,45	0,1317	01-089-8934, 01-074-1867, 01-078-4614
70,8870	1,54252	29474,29	11,74	0,1495	01-089-8934, 01-083-1604
71,7072	1,52720	208,89	0,08	0,0780	01-078-4614
72,9232	1,50518	140,12	0,06	0,0780	01-083-1604
73,9604	1,48703	122,60	0,05	0,0780	00-041-1480, 01-078-4339, 01-083-1604
75,9592	1,45358	3920,88	1,56	0,1583	01-089-8934, 00-041-1480, 01-083-1604
76,7122	1,44148	300,41	0,12	0,1164	01-078-4614, 01-083-1604
77,1484	1,43459	0,00	0,00	0,0780	01-074-1867, 01-078-4339, 01-083-1604
78,1688	1,41880	1400,49	0,56	0,1120	01-089-8934, 01-074-1867, 01-078-4614, 01-083-1604
80,6095	1,38286	7041,91	2,81	0,1756	01-089-8934, 00-041-1480
81,1315	1,37548	9035,95	3,60	0,1331	01-089-8934, 01-078-4339
81,2449	1,37390	5739,48	2,29	0,2571	01-089-8934
82,7073	1,35387	3359,95	1,34	0,0952	00-041-1480, 01-074-1867, 01-078-4614

83,6210	1,34175	424,84	0,17	0,2664	00-041-1480, 01-078-4339, 01-078-4614
86,7661	1,30229	1055,59	0,42	0,1969	01-078-4339
87,8211	1,28978	32,44	0,01	1,8509	01-089-8934, 01-074-1867
87,9061	1,28878	2279,07	0,91	0,1773	01-089-8934, 01-074-1867

Pattern List:

Ref.Code	Compound Name	Mineral Name	Chem. Formula	SemiQuant[%]
01-089-8934	Silicon Oxide	Quartz	Si O2	85
00-041-1480	Sodium Calcium Aluminum Silicate	Albite	(Na , Ca) Al (Si , Al)3 O8	3
01-074-1867	Calcium Carbonate	Vaterite, syn	Ca (C O3)	stopy
01-078-4339	Calcium Carbonate	Aragonite	Ca (C O3)	Stopy možné
01-078-4614	Calcium Carbonate	Calcite, syn	Ca (C O3)	5
01-083-1604	Potassium Aluminum Silicate	Microcline	K (Al Si3 O8)	2
