# Supplementary material for: Corrosion Response of Steel to Penetration of Chlorides in DC-Treated Hardened Portland Cement Mortar
Source: Materials (Basel). 2025 Jul 17;18(14):3365. doi: 10.3390/ma18143365 (PMC12300289; doi:10.3390/ma18143365)
Supplement: Supplementary file 1 [file materials-18-03365-s001.zip › protocol s2.pdf]

Protocol S2

Measurement Conditions:

Dataset Name XADS20_5-90_T120_s6_sp0_rp0_MA-11
File name \\share\rentgenka980\PC2_XPERT\2024\Kouril\2024-04-23\XADS20_5-90_T120_s6_sp0_rp0_MA-11.xrdml
Sample Identification MA-11
17min50s
PHD Lower Level = 4.02 (keV), PHD Upper Level = 9.70 (keV)

Measurement Start Date/Time	23.04.2024 11:52:01
Operator	localadmin
Raw Data Origin	XRD measurement (*.XRDML)
Scan Axis	Gonio
Start Position [°2θ]	4,8647
End Position [°2θ]	89,8067
Step Size [°2θ]	0,0390
Scan Step Time [s]	116,5350
Scan Type	Continuous
PSD Mode	Scanning
PSD Length [°2θ]	3,35
Offset [°2θ]	0,0000
Divergence Slit Type	Fixed
Divergence Slit Size [°]	1,0000
Specimen Length [mm]	20,00
Measurement Temperature [°C]	25,00
Anode Material	Co
Intended Wavelength Type	K-α1
K-α1 [Å]	1,78901
K-α2 [Å]	1,79290
K-β1 [Å]	1,62083
K-β2 [Å]	1,38113
K-β3 [Å]	1,39261
K-A2 / K-A1 Ratio	0,50000
K-Alpha2 Line Shift	0,00000
K Absorption Edge	1,37868
Generator Settings	40 mA, 35 kV
Diffractometer Type	0000000080910230
Diffractometer Number	0
Goniometer Radius [mm]	240,00
Dist. Focus-Diverg. Slit [mm]	100,00
Incident Beam Monochromator	No
Spinning	No
Fast detector	PIXcel1D_1D detector

Main Graphics, Analyze View:

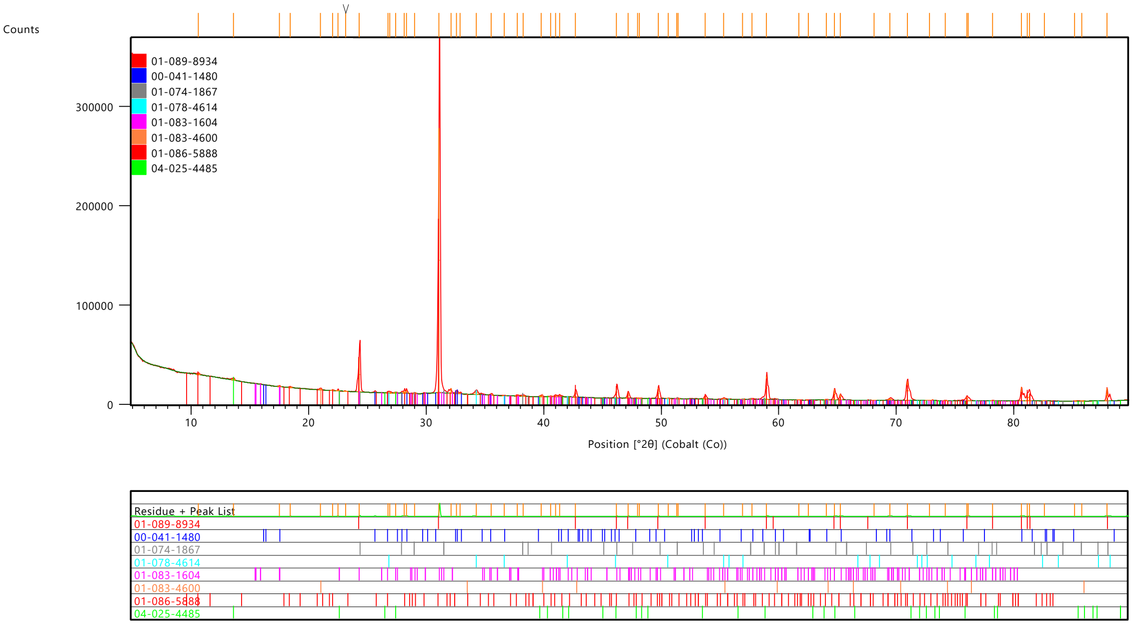

Peak List:

Pos. [°2θ]	d-spacing [Å]	Height [cts]	Rel. Int. [%]	FWHM Left [°2θ]	Matched by
10,6014	9,68259	1077,82	0,40	0,2304	01-086-5888
13,5731	7,56960	1447,81	0,54	0,2492	04-025-4485
17,4881	5,88411	756,71	0,28	0,1651	00-041-1480, 01-083-1604
18,4307	5,58558	693,14	0,26	0,2301	01-086-5888
20,9833	4,91236	1524,50	0,57	0,1437	01-083-4600
22,0372	4,68015	760,08	0,29	0,0780	01-086-5888
22,4854	4,58802	863,78	0,32	0,0780	01-083-1604, 04-025-4485
23,1272	4,46236	0,00	0,00	0,0780	
24,3038	4,24935	34370,38	12,89	0,1694	01-089-8934, 01-074-1867, 01-083-1604
26,7410	3,86818	1014,78	0,38	0,1599	00-041-1480, 01-078-4614, 01-083-1604, 01-086-5888
26,9037	3,84521	700,89	0,26	0,2274	01-078-4614
27,3928	3,77784	622,48	0,23	0,1811	00-041-1480, 01-083-1604, 04-025-4485
28,1071	3,68368	2164,44	0,81	0,3171	00-041-1480, 01-086-5888
28,3127	3,65748	2782,10	1,04	0,0780	00-041-1480, 01-086-5888

28,9908	3,57370	844,70	0,32	0,1298	01-074-1867, 01-083-1604, 01-086-5888
31,0905	3,33771	266565,80	100,00	0,1412	01-089-8934, 01-083-1604, 01-086-5888
32,1003	3,23535	5177,37	1,94	0,0900	01-083-1604, 01-086-5888
32,5712	3,18982	2669,15	1,00	0,1532	00-041-1480, 01-086-5888
32,8855	3,16015	515,51	0,19	0,0780	00-041-1480
34,2585	3,03707	3241,30	1,22	0,2839	01-078-4614
35,5251	2,93210	1176,36	0,44	0,1810	00-041-1480, 01-083-1604
36,6278	2,84672	497,62	0,19	0,0877	00-041-1480, 01-078-4614
37,7330	2,76625	717,65	0,27	0,3673	01-083-1604, 01-086-5888
38,2365	2,73116	884,46	0,33	0,3273	01-074-1867
39,7613	2,63042	1175,20	0,44	0,2332	00-041-1480, 01-083-4600, 04-025-4485
40,5696	2,58015	691,27	0,26	0,0780	01-074-1867, 01-083-1604, 01-086-5888
41,0009	2,55416	1323,10	0,50	0,1846	01-083-1604, 01-086-5888
41,3585	2,53303	1362,69	0,51	0,2363	00-041-1480, 01-083-1604
42,7106	2,45642	6793,74	2,55	0,1423	01-089-8934, 01-083-1604, 01-083-4600
46,2014	2,27987	11641,91	4,37	0,1361	01-089-8934, 00-041-1480, 01-074-1867, 01-078-4614, 01-083-1604, 01-086-5888, 04-025-4485
47,1747	2,23544	6044,73	2,27	0,1411	01-089-8934, 01-083-1604, 01-086-5888
47,9893	2,19969	403,25	0,15	0,1930	00-041-1480, 01-083-1604, 01-086-5888, 04-025-4485
48,1253	2,19384	59,20	0,02	0,1583	01-083-1604
49,7532	2,12641	10178,57	3,82	0,1680	01-089-8934, 00-041-1480, 01-074-1867,

					01-083-1604, 01-086-5888
50,6064	2,09286	428,41	0,16	0,1090	01-078-4614
51,3080	2,06614	493,23	0,19	0,0960	01-074-1867, 01-083-1604
51,4322	2,06149	478,05	0,18	0,1337	01-074-1867, 01-086-5888
53,7310	1,97944	3165,07	1,19	0,1845	01-089-8934, 01-074-1867, 01-086-5888
55,2960	1,92765	1210,58	0,45	0,2398	01-078-4614, 01-083-4600
56,9223	1,87699	343,96	0,13	0,1120	00-041-1480, 01-078-4614, 01-083-1604, 01-086-5888
57,7098	1,85354	532,67	0,20	0,1870	00-041-1480, 01-074-1867, 01-083-1604
58,9704	1,81737	22913,25	8,60	0,1404	01-089-8934, 04-025-4485
61,6949	1,74451	245,88	0,09	0,1311	01-074-1867, 01-083-1604
62,5305	1,72351	251,26	0,09	0,0935	00-041-1480, 01-083-1604, 01-086-5888
64,0561	1,68668	600,38	0,23	0,1224	00-041-1480, 01-083-1604, 01-083-4600, 01-086-5888, 04-025-4485
64,7322	1,67095	10654,48	4,00	0,1563	01-089-8934, 01-074-1867, 01-083-1604, 01-086-5888, 04-025-4485
65,2458	1,65923	5774,01	2,17	0,1532	01-089-8934, 00-041-1480, 01-083-1604, 01-086-5888
68,0981	1,59761	154,49	0,06	0,4872	01-083-1604, 01-086-5888
69,4613	1,57008	2030,60	0,76	0,2775	00-041-1480, 01-074-1867, 01-083-1604, 01-086-5888
70,9565	1,54120	19109,41	7,17	0,1959	01-089-8934, 01-083-1604, 01-086-5888

72,8256	1,50692	257,35	0,10	0,7940	01-078-4614, 01-083-1604, 01-086-5888
74,1597	1,48360	10,86	0,00	0,0780	01-086-5888
75,9968	1,45297	4102,08	1,54	0,2215	01-089-8934, 01-083-1604, 01-086-5888, 04-025-4485
76,1308	1,45080	0,00	0,00	0,3312	01-089-8934, 01-086-5888
78,2016	1,41830	640,75	0,24	0,1692	01-089-8934, 01-074-1867, 01-083-1604
80,6552	1,38221	13522,98	5,07	0,1505	01-089-8934, 00-041-1480
81,1651	1,37501	6568,55	2,46	0,1987	01-089-8934
81,3378	1,37260	7393,53	2,77	0,1720	01-089-8934
82,6076	1,35521	75,18	0,03	0,1760	00-041-1480, 01-078-4614, 01-086-5888
85,1917	1,32162	274,68	0,10	0,1348	00-041-1480
85,7817	1,31428	184,03	0,07	0,2108	01-074-1867
87,9413	1,28837	12822,20	4,81	0,1405	01-089-8934, 01-074-1867

Pattern List:

Ref.Code	Compound Name	Mineral Name	Chem. Formula	SemiQuant[%]
01-089-8934	Silicon Oxide	Quartz	Si O2	80
00-041-1480	Sodium Calcium Aluminum Silicate	Albite	(Na , Ca) Al (Si , Al)3 O8	5
01-074-1867	Calcium Carbonate	Vaterite, syn	Ca (C O3)	3
01-078-4614	Calcium Carbonate	Calcite, syn	Ca (C O3)	3
01-083-1604	Potassium Aluminum Silicate	Microcline	K (Al Si3 O8)	3
01-083-4600	Calcium Hydroxide	Portlandite, syn	Ca (O H)2	1
01-086-5888	Calcium Aluminum Carbonate Sulfate Hydroxide Hydrate	Ettringite	Ca6 Al2 ((S O4)2.81 (C O3)0.51) (O H)12 (H2 O)24.4	možné
04-025-4485	Magnesium Aluminum Carbonate Hydroxide Hydrate	Quintinite,	Mg2 Al (C O3)0.5 (O H)6 (H2 O)1.5	možné
