# Supplementary material for: Corrosion Response of Steel to Penetration of Chlorides in DC-Treated Hardened Portland Cement Mortar
Source: Materials (Basel). 2025 Jul 17;18(14):3365. doi: 10.3390/ma18143365 (PMC12300289; doi:10.3390/ma18143365)
Supplement: Supplementary file 1 [file materials-18-03365-s001.zip › protocol s3.pdf]

Protocol S3

Measurement Conditions:

Dataset Name XADS20_5-90_T120_s6_sp0_rp0_MA-9
File name \\share\rentgenka980\PC2_XPERT\2024\Kouril\2024-04-23\XADS20_5-90_T120_s6_sp0_rp0_MA-9.xrdml
Sample Identification MA-9
17min50s
PHD Lower Level = 4.02 (keV), PHD Upper Level = 9.70 (keV)
Measurement Start Date/Time 23.04.2024 11:30:49
Operator localadmin
Raw Data Origin XRD measurement (*.XRDML)
Scan Axis Gonio
Start Position [°2θ] 4,8147
End Position [°2θ] 89,7567
Step Size [°2θ] 0,0390
Scan Step Time [s] 116,5350
Scan Type Continuous
PSD Mode Scanning
PSD Length [°2θ] 3,35
Offset [°2θ] 0,0000
Divergence Slit Type Fixed
Divergence Slit Size [°] 1,0000
Specimen Length [mm] 20,00
Measurement Temperature [°C] 25,00
Anode Material Co
Intended Wavelength Type K-α1
K-α1 [Å] 1,78901
K-α2 [Å] 1,79290
K-β1 [Å] 1,62083
K-β2 [Å] 1,38113
K-β3 [Å] 1,39261
K-A2 / K-A1 Ratio 0,50000
K-Alpha2 Line Shift 0,00000
K Absorption Edge 1,37868
Generator Settings 40 mA, 35 kV
Diffractometer Type 0000000080910230
Diffractometer Number 0
Goniometer Radius [mm] 240,00
Dist. Focus-Diverg. Slit [mm] 100,00
Incident Beam Monochromator No
Spinning No
Fast detector PIXcel1D_1D detector

Main Graphics, Analyze View:

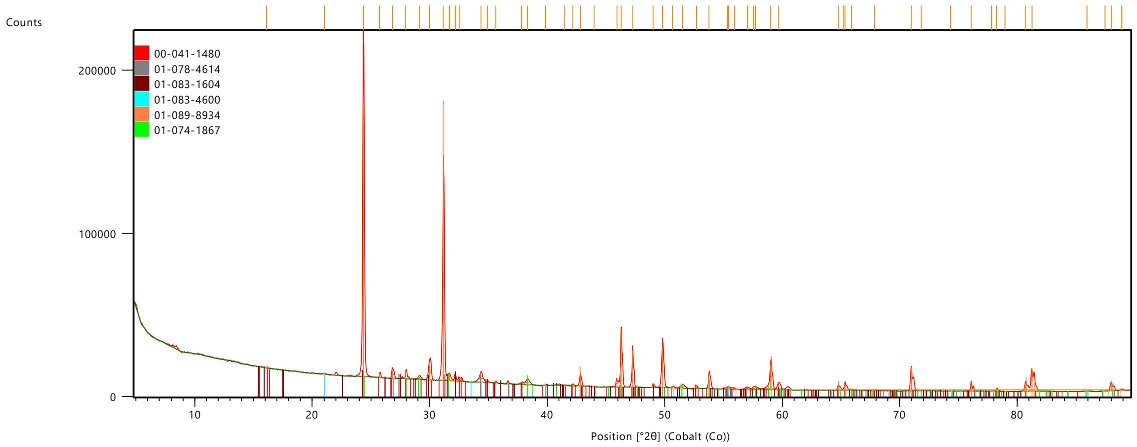

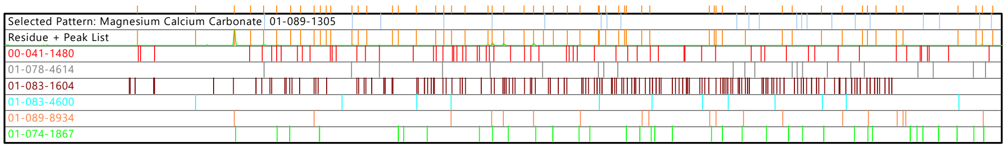

Peak List:

Pos. [°2θ]	d-spacing [Å]	Height [cts]	Rel. Int. [%]	FWHM Left [°2θ]	Matched by
16,1180	6,38055	348,57	0,21	0,3731	00-041-1480
21,0653	4,89347	476,21	0,29	0,1094	01-083-4600
24,3525	4,24098	166616,20	100,00	0,1339	01-083-1604, 01-089-8934, 01-074-1867
25,7434	4,01540	2394,13	1,44	0,0780	00-041-1480
26,8579	3,85165	4943,68	2,97	0,2096	00-041-1480, 01-078-4614, 01-083-1604
27,9669	3,70179	5620,67	3,37	0,0780	00-041-1480, 01-074-1867
29,1342	3,55648	1699,58	1,02	0,2724	01-083-1604, 01-074-1867
29,9988	3,45624	8936,36	5,36	0,2038	00-041-1480, 01-083-1604
31,1568	3,33079	107693,20	64,64	0,1274	01-083-1604, 01-089-8934
31,6653	3,27864	3636,63	2,18	0,1754	01-083-1604, 01-074-1867
32,1702	3,22851	4050,00	2,43	0,0803	01-083-1604
32,5510	3,19174	1462,98	0,88	0,3636	00-041-1480
34,3614	3,02825	4654,57	2,79	0,2903	01-078-4614
34,9062	2,98243	832,69	0,50	0,0780	00-041-1480, 01-083-1604

35,6214	2,92443	1085,33	0,65	0,1071	00-041-1480, 01-083-1604
37,8285	2,75952	907,32	0,54	0,2268	01-083-1604
38,3258	2,72503	2548,71	1,53	0,3365	01-074-1867
39,8605	2,62413	702,44	0,42	0,0780	01-083-1604, 01-083-4600
41,4867	2,52555	0,00	0,00	0,0810	00-041-1480
42,1819	2,48578	759,64	0,46	0,0780	00-041-1480, 01-078-4614, 01-083-1604
42,8172	2,45059	6615,68	3,97	0,1381	00-041-1480, 01-083-1604, 01-083-4600, 01-089-8934
43,9806	2,38885	250,34	0,15	0,1110	00-041-1480, 01-083-1604
45,9318	2,29253	4498,21	2,70	0,1154	00-041-1480
46,2808	2,27618	35330,87	21,20	0,0982	00-041-1480, 01-078-4614, 01-083-1604, 01-089-8934, 01-074-1867
47,2696	2,23121	22480,04	13,49	0,0973	01-083-1604, 01-089-8934
49,0133	2,15648	2435,40	1,46	0,0780	00-041-1480, 01-083-1604
49,8081	2,12421	24310,83	14,59	0,1595	01-083-1604, 01-089-8934
50,6619	2,09072	955,81	0,57	0,1544	01-078-4614
51,5111	2,05855	1550,05	0,93	0,4189	01-074-1867
52,6717	2,01632	2304,38	1,38	0,0810	00-041-1480, 01-083-1604
53,7823	1,97769	9563,34	5,74	0,1358	01-083-1604, 01-089-8934, 01-074-1867
55,3513	1,92588	776,68	0,47	0,1614	01-078-4614, 01-083-1604, 01-083-4600
55,4293	1,92338	383,99	0,23	0,5494	01-078-4614, 01-083-1604, 01-083-4600
55,9334	1,90743	90,42	0,05	0,5897	01-078-4614
57,0437	1,87333	951,40	0,57	0,1662	00-041-1480, 01-078-4614, 01-083-1604
57,5488	1,85828	827,81	0,50	0,1088	01-083-1604, 01-074-1867
57,7168	1,85333	841,49	0,51	0,2662	00-041-1480, 01-083-1604, 01-074-1867

59,0215	1,81593	14873,00	8,93	0,1618	01-089-8934, 01-074-1867
59,6943	1,79732	4106,23	2,46	0,1237	00-041-1480, 01-083-1604, 01-083-4600, 01-089-8934, 01-074-1867
64,7743	1,66998	3506,37	2,10	0,1516	01-083-1604, 01-089-8934, 01-074-1867
65,1996	1,66028	297,11	0,18	0,0780	00-041-1480, 01-083-1604, 01-089-8934, 01-074-1867
65,3410	1,65708	4829,93	2,90	0,1290	00-041-1480, 01-083-1604, 01-089-8934
65,8952	1,64470	525,69	0,32	0,4402	01-083-1604, 01-074-1867
67,8285	1,60319	471,39	0,28	0,3377	01-078-4614, 01-083-1604, 01-089-8934, 01-074-1867
70,9956	1,54047	14494,11	8,70	0,1426	01-083-1604, 01-089-8934
71,8397	1,52476	188,88	0,11	0,4162	01-078-4614, 01-083-1604
74,3164	1,48092	189,46	0,11	0,2092	01-083-4600
76,0724	1,45174	5931,08	3,56	0,1099	01-083-1604, 01-089-8934
77,8064	1,42436	267,68	0,16	0,2244	01-078-4614
78,2445	1,41765	1850,86	1,11	0,1418	01-078-4614, 01-083-1604, 01-089-8934
78,9573	1,40692	28,77	0,02	0,0780	01-083-1604
80,6894	1,38172	5071,60	3,04	0,1723	00-041-1480, 01-089-8934
81,2392	1,37398	14289,33	8,58	0,1762	01-089-8934
85,9368	1,31237	273,63	0,16	0,5929	01-083-4600, 01-074-1867
87,4809	1,29377	719,48	0,43	0,1487	01-078-4614, 01-074-1867
87,9879	1,28783	5663,23	3,40	0,1867	01-089-8934, 01-074-1867

Pattern List:

Ref.Code	Compound Name	Mineral Name	Chem. Formula	SemiQuant[%]
00-041-1480	Sodium Calcium	Albite	(Na , Ca) Al (	4

	Aluminum Silicate		Si , Al)3 O8	
01-078-4614	Calcium Carbonate	Calcite, syn	Ca (C O3)	6
01-083-1604	Potassium Aluminum Silicate	Microcline	K (Al Si3 O8)	6
01-083-4600	Calcium Hydroxide	Portlandite, syn	Ca (O H)2	stopy
01-089-8934	Silicon Oxide	Quartz	Si O2	75
01-074-1867	Calcium Carbonate	Vaterite, syn	Ca (C O3)	8
