# Supplementary material for: Corrosion Response of Steel to Penetration of Chlorides in DC-Treated Hardened Portland Cement Mortar
Source: Materials (Basel). 2025 Jul 17;18(14):3365. doi: 10.3390/ma18143365 (PMC12300289; doi:10.3390/ma18143365)
Supplement: Supplementary file 1 [file materials-18-03365-s001.zip › protocol s4.pdf]

Protocol S4

Measurement Conditions:

Dataset Name XADS20_5-90_T120_s6_sp0_rp0_MA-8
File name \\share\rentgenka980\PC2_XPERT\2024\Kouril\2024-04-23\XADS20_5-90_T120_s6_sp0_rp0_MA-8.xrdml
Sample Identification MA-8
17min50s
PHD Lower Level = 4.02 (keV), PHD Upper Level = 9.70 (keV)

Measurement Start Date/Time	24.04.2024 11:18:55
Operator	localadmin
Raw Data Origin	XRD measurement (*.XRDML)
Scan Axis	Gonio
Start Position [°2θ]	4,8647
End Position [°2θ]	89,8067
Step Size [°2θ]	0,0390
Scan Step Time [s]	116,5350
Scan Type	Continuous
PSD Mode	Scanning
PSD Length [°2θ]	3,35
Offset [°2θ]	0,0000
Divergence Slit Type	Fixed
Divergence Slit Size [°]	1,0000
Specimen Length [mm]	20,00
Measurement Temperature [°C]	25,00
Anode Material	Co
Intended Wavelength Type	K-α1
K-α1 [Å]	1,78901
K-α2 [Å]	1,79290
K-β1 [Å]	1,62083
K-β2 [Å]	1,38113
K-β3 [Å]	1,39261
K-A2 / K-A1 Ratio	0,50000
K-Alpha2 Line Shift	0,00000
K Absorption Edge	1,37868
Generator Settings	40 mA, 35 kV
Diffractometer Type	0000000080910230
Diffractometer Number	0
Goniometer Radius [mm]	240,00
Dist. Focus-Diverg. Slit [mm]	100,00
Incident Beam Monochromator	No
Spinning	No
Fast detector	PIXcel1D_1D detector

Main Graphics, Analyze View:

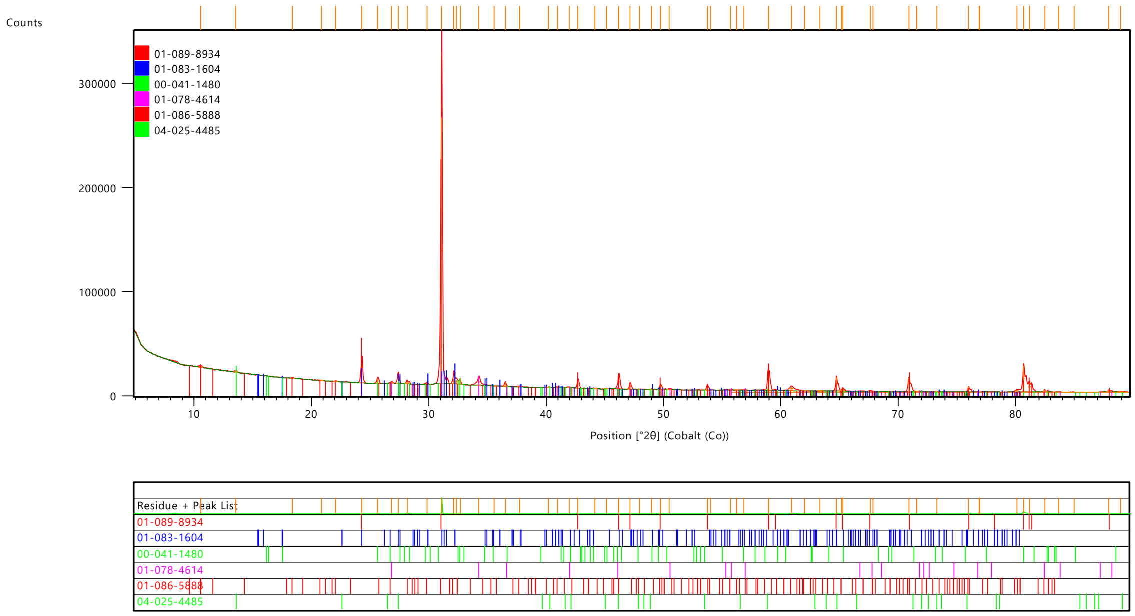

Peak List:

Pos. [°2θ]	d-spacing [Å]	Height [cts]	Rel. Int. [%]	FWHM Left [°2θ]	Matched by
10,5587	9,72167	1121,29	0,44	0,3053	01-086-5888
13,5471	7,58407	1034,43	0,40	0,2671	04-025-4485
18,3967	5,59582	609,99	0,24	0,0965	01-086-5888
20,8522	4,94290	342,58	0,13	0,2027	01-086-5888
22,0534	4,67674	411,17	0,16	0,2021	01-086-5888
24,2788	4,25366	17443,45	6,82	0,1536	01-089-8934, 01-083-1604
25,6316	4,03262	4426,91	1,73	0,1815	00-041-1480, 01-086-5888
26,8116	3,85819	1297,27	0,51	0,2578	01-083-1604, 00-041-1480, 01-078-4614, 01-086-5888
27,3976	3,77719	8077,74	3,16	0,1622	01-083-1604, 00-041-1480, 04-025-4485
28,1712	3,67547	2012,69	0,79	0,3148	00-041-1480, 01-086-5888
29,8424	3,47394	2082,93	0,81	0,0780	01-083-1604, 00-041-1480, 01-086-5888
31,0714	3,33972	255742,80	100,00	0,1225	01-089-8934, 01-083-1604, 01-086-5888

32,1295	3,23248	5045,29	1,97	0,1013	01-083-1604, 01-086-5888
32,3257	3,21339	3736,22	1,46	0,5470	01-083-1604, 01-086-5888
32,6901	3,17852	5900,34	2,31	0,0900	00-041-1480
34,2629	3,03670	6302,14	2,46	0,2350	01-078-4614
35,5675	2,92872	588,40	0,23	0,1013	01-083-1604, 00-041-1480, 01-086-5888
36,5058	2,85591	3670,51	1,44	0,1250	00-041-1480, 01-078-4614
37,7325	2,76628	660,11	0,26	0,4568	01-083-1604, 01-086-5888
40,2169	2,60183	420,97	0,16	0,0780	01-086-5888, 04-025-4485
40,9723	2,55587	865,26	0,34	0,2184	01-086-5888
41,9834	2,49699	828,50	0,32	0,1631	00-041-1480, 01-078-4614, 01-086-5888, 04-025-4485
42,7066	2,45663	7445,78	2,91	0,1437	01-089-8934, 01-083-1604
44,1540	2,37994	533,29	0,21	0,1454	01-083-1604, 00-041-1480, 01-086-5888
45,1539	2,32990	404,70	0,16	0,0780	01-083-1604, 00-041-1480, 04-025-4485
46,1661	2,28152	13474,24	5,27	0,1413	01-089-8934, 01-083-1604, 01-078-4614, 01-086-5888, 04-025-4485
47,1481	2,23663	5020,64	1,96	0,1552	01-089-8934, 01-083-1604, 01-086-5888
47,9352	2,20202	301,51	0,12	0,0878	01-083-1604, 00-041-1480, 01-086-5888, 04-025-4485
48,9936	2,15729	665,13	0,26	0,0871	01-083-1604, 00-041-1480, 01-086-5888, 04-025-4485
49,7343	2,12716	4092,45	1,60	0,1659	01-089-8934, 01-083-1604, 00-041-1480, 01-086-5888
50,4806	2,09773	451,34	0,18	0,3734	00-041-1480, 01-078-4614

53,7226	1,97973	4662,43	1,82	0,1426	01-089-8934, 01-086-5888, 04-025-4485
54,0218	1,96958	1533,60	0,60	0,0780	01-083-1604, 01-086-5888
55,7054	1,91461	471,15	0,18	0,2071	01-083-1604, 01-078-4614, 01-086-5888
56,2246	1,89835	118,59	0,05	0,3251	01-086-5888
56,8577	1,87895	471,18	0,18	0,2796	01-083-1604, 00-041-1480, 01-078-4614, 01-086-5888
58,9334	1,81841	19102,13	7,47	0,1742	01-089-8934, 01-083-1604, 04-025-4485
60,8997	1,76506	3460,89	1,35	0,3397	01-086-5888
64,7244	1,67113	13288,63	5,20	0,1316	01-089-8934, 01-083-1604, 01-086-5888, 04-025-4485
65,1761	1,66081	577,53	0,23	0,1192	01-089-8934, 01-083-1604, 00-041-1480, 01-086-5888
65,2694	1,65870	2795,60	1,09	0,1452	01-089-8934, 01-083-1604, 00-041-1480, 01-086-5888
67,6083	1,60779	598,96	0,23	0,1845	01-089-8934, 01-078-4614
67,8306	1,60315	160,15	0,06	0,0780	01-089-8934, 01-078-4614, 01-086-5888
70,9302	1,54170	13291,74	5,20	0,1661	01-089-8934, 01-083-1604, 01-086-5888
71,5689	1,52975	218,75	0,09	0,2306	00-041-1480, 01-086-5888
73,2898	1,49870	95,12	0,04	0,2626	01-083-1604, 00-041-1480, 01-086-5888
75,9876	1,45312	4949,76	1,94	0,1378	01-089-8934, 01-083-1604, 00-041-1480, 01-086-5888, 04-025-4485
76,9207	1,43817	369,35	0,14	0,0780	01-083-1604, 01-078-4614
80,1123	1,38998	1732,42	0,68	0,3154	01-083-1604,

					01-086-5888
80,6825	1,38182	25211,84	9,86	0,1640	01-089-8934, 00-041-1480
81,1734	1,37490	9384,08	3,67	0,2317	01-089-8934
82,5047	1,35659	2520,02	0,99	0,1094	00-041-1480, 01-078-4614, 01-086-5888
83,6716	1,34109	197,93	0,08	0,1847	00-041-1480, 01-078-4614
85,0059	1,32396	77,27	0,03	0,1587	00-041-1480
87,9559	1,28820	2637,71	1,03	0,1604	01-089-8934, 01-078-4614
88,9296	1,27701	532,89	0,21	0,5448	04-025-4485

Pattern List:

Ref.Code	Compound Name	Mineral Name	Chem. Formula	SemiQuant[%]
01-089-8934	Silicon Oxide	Quartz	Si O2	80
01-083-1604	Potassium Aluminum Silicate	Microcline	K (Al Si3 O8)	7
00-041-1480	Sodium Calcium Aluminum Silicate	Albite,	(Na , Ca) Al (Si , Al)3 O8	5
01-078-4614	Calcium Carbonate	Calcite, syn	Ca (C O3)	5
01-086-5888	Calcium Aluminum Carbonate Sulfate Hydroxide Hydrate	Ettringite	Ca6 Al2 ((S O4)2.81 (C O3)0.51) (O H)12 (H2 O)24.4	2
04-025-4485	Magnesium Aluminum Carbonate Hydroxide Hydrate	Quintinite,	Mg2 Al (C O3)0.5 (O H)6 (H2 O)1.5	1
