# Supplementary material for: Corrosion Response of Steel to Penetration of Chlorides in DC-Treated Hardened Portland Cement Mortar
Source: Materials (Basel). 2025 Jul 17;18(14):3365. doi: 10.3390/ma18143365 (PMC12300289; doi:10.3390/ma18143365)
Supplement: Supplementary file 1 [file materials-18-03365-s001.zip › protocol s5.pdf]

# Protocol S5

## *Measurement Conditions:*

Dataset Name XADS20_5-90_T120_s6_sp0_rp0_MA-12
File name \\share\rentgenka980\PC2_XPERT\2024\Kouril\2024-04-23\XADS20_5-90_T120_s6_sp0_rp0_MA-12.xrdml
Sample Identification MA-12
17min50s
PHD Lower Level = 4.02 (keV), PHD Upper Level = 9.70 (keV)

| | |
|---|---|
| Measurement Start Date/Time | 24.04.2024 10:58:05 |
| Operator | localadmin |
| Raw Data Origin | XRD measurement (*.XRDML) |
| Scan Axis | Gonio |
| Start Position [°2θ] | 4,8147 |
| End Position [°2θ] | 89,7567 |
| Step Size [°2θ] | 0,0390 |
| Scan Step Time [s] | 116,5350 |
| Scan Type | Continuous |
| PSD Mode | Scanning |
| PSD Length [°2θ] | 3,35 |
| Offset [°2θ] | 0,0000 |
| Divergence Slit Type | Fixed |
| Divergence Slit Size [°] | 1,0000 |
| Specimen Length [mm] | 20,00 |
| Measurement Temperature [°C] | 25,00 |
| Anode Material | Co |
| Intended Wavelength Type | K-α1 |
| K-α1 [Å] | 1,78901 |
| K-α2 [Å] | 1,79290 |
| K-β1 [Å] | 1,62083 |
| K-β2 [Å] | 1,38113 |
| K-β3 [Å] | 1,39261 |
| K-A2 / K-A1 Ratio | 0,50000 |
| K-Alpha2 Line Shift | 0,00000 |
| K Absorption Edge | 1,37868 |
| Generator Settings | 40 mA, 35 kV |
| Diffractometer Type | 0000000080910230 |
| Diffractometer Number | 0 |
| Goniometer Radius [mm] | 240,00 |
| Dist. Focus-Diverg. Slit [mm] | 100,00 |
| Incident Beam Monochromator | No |
| Spinning | No |
| Fast detector | PIXcel1D_1D detector |

## Main Graphics, Analyze View:

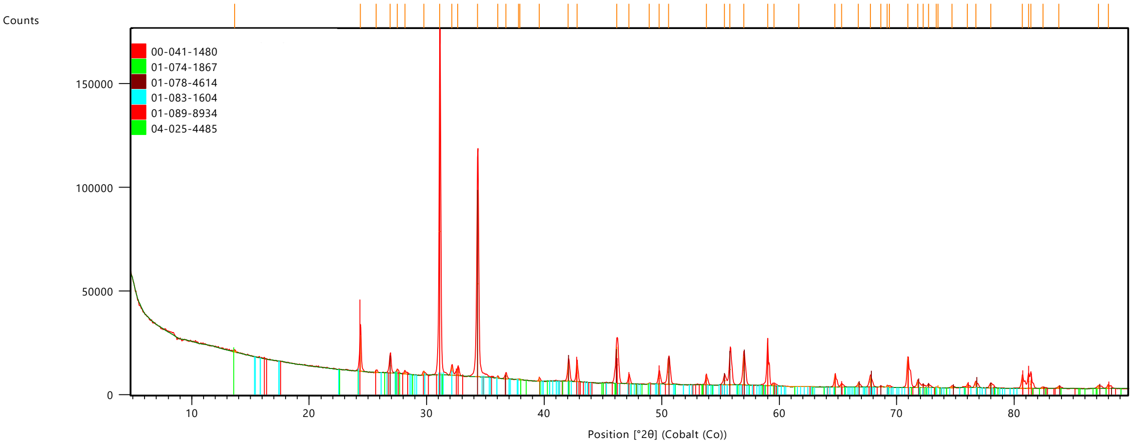


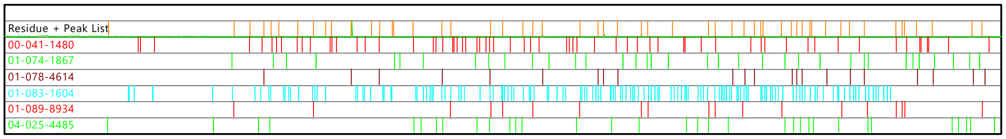


## Peak List:

| Pos. [°2θ] | d-spacing [Å] | Height [cts] | Rel. Int. [%] | FWHM Left [°2θ] | Matched by |
|---|---|---|---|---|---|
| 13,6582 | 7,52265 | 679,31 | 0,49 | 0,1735 | 04-025-4485 |
| 24,3484 | 4,24168 | 17661,52 | 12,75 | 0,1130 | 01-074-1867, 01-083-1604, 01-089-8934 |
| 25,7089 | 4,02070 | 1178,42 | 0,85 | 0,0780 | 00-041-1480 |
| 26,8883 | 3,84738 | 7126,75 | 5,15 | 0,1511 | 00-041-1480, 01-078-4614 |
| 27,5030 | 3,76298 | 1960,41 | 1,42 | 0,1012 | 00-041-1480, 01-074-1867, 01-083-1604, 04-025-4485 |
| 28,1483 | 3,67840 | 1078,79 | 0,78 | 0,1882 | 00-041-1480 |
| 29,7536 | 3,48407 | 1990,24 | 1,44 | 0,0786 | 00-041-1480, 01-083-1604 |
| 31,1045 | 3,33625 | 138506,20 | 100,00 | 0,1192 | 01-074-1867, 01-083-1604, 01-089-8934 |
| 32,1495 | 3,23053 | 4002,05 | 2,89 | 0,1469 | 01-083-1604 |
| 32,6213 | 3,18504 | 2947,48 | 2,13 | 0,2458 | 00-041-1480 |
| 34,3344 | 3,03057 | 89889,19 | 64,90 | 0,1401 | 01-078-4614 |
| 36,0393 | 2,89163 | 922,37 | 0,67 | 0,0780 | 01-083-1604 |
| 36,7386 | 2,83843 | 2182,40 | 1,58 | 0,1616 | 00-041-1480, 01-078-4614 |

| | | | | | |
|---|---|---|---|---|---|
| 37,8072 | 2,76101 | 322,82 | 0,23 | 0,0780 | 01-074-1867, 01-083-1604 |
| 37,9464 | 2,75126 | 177,42 | 0,13 | 0,0900 | 01-074-1867, 01-083-1604 |
| 39,5683 | 2,64273 | 1761,60 | 1,27 | 0,0963 | 00-041-1480, 04-025-4485 |
| 42,0488 | 2,49329 | 8582,11 | 6,20 | 0,1651 | 00-041-1480, 01-078-4614, 01-083-1604, 04-025-4485 |
| 42,7795 | 2,45264 | 8497,92 | 6,14 | 0,1275 | 01-083-1604, 01-089-8934 |
| 46,1596 | 2,28183 | 17552,57 | 12,67 | 0,2196 | 01-074-1867, 01-078-4614, 01-083-1604, 01-089-8934, 04-025-4485 |
| 47,1990 | 2,23436 | 4200,70 | 3,03 | 0,0780 | 01-074-1867, 01-083-1604, 01-089-8934 |
| 48,9415 | 2,15945 | 562,26 | 0,41 | 0,0780 | 00-041-1480, 01-083-1604, 04-025-4485 |
| 49,7726 | 2,12563 | 5594,25 | 4,04 | 0,1562 | 00-041-1480, 01-074-1867, 01-083-1604, 01-089-8934 |
| 50,5767 | 2,09401 | 11492,35 | 8,30 | 0,1623 | 01-078-4614 |
| 53,7854 | 1,97759 | 4620,71 | 3,34 | 0,1341 | 00-041-1480, 01-083-1604, 01-089-8934 |
| 55,3210 | 1,92685 | 3859,51 | 2,79 | 0,2306 | 01-078-4614, 01-083-1604 |
| 55,8124 | 1,91123 | 15049,90 | 10,87 | 0,1822 | 01-078-4614 |
| 56,9898 | 1,87496 | 14003,81 | 10,11 | 0,1844 | 00-041-1480, 01-078-4614, 01-083-1604 |
| 59,0088 | 1,81629 | 20170,63 | 14,56 | 0,1207 | 01-083-1604, 01-089-8934, 04-025-4485 |
| 59,5692 | 1,80075 | 912,14 | 0,66 | 0,2169 | 00-041-1480, 01-074-1867, 01-083-1604, 01-089-8934 |
| 61,6698 | 1,74515 | 64,04 | 0,05 | 2,7258 | 01-083-1604 |
| 64,7393 | 1,67079 | 5829,33 | 4,21 | 0,1558 | 01-074-1867, 01-083-1604, 01-089-8934, 04-025-4485 |
| 65,2996 | 1,65802 | 1635,31 | 1,18 | 0,1673 | 00-041-1480, |

| | | | | | |
|---|---|---|---|---|---|
| | | | | | 01-074-1867, 01-083-1604, 01-089-8934 |
| 66,7195 | 1,62669 | 1963,15 | 1,42 | 0,1152 | 01-078-4614, 01-083-1604 |
| 67,7442 | 1,60495 | 4776,62 | 3,45 | 0,2162 | 01-078-4614, 01-083-1604, 01-089-8934 |
| 68,6473 | 1,58638 | 316,42 | 0,23 | 0,1047 | 01-078-4614 |
| 69,1877 | 1,57551 | 911,03 | 0,66 | 0,0780 | 00-041-1480, 01-074-1867, 01-083-1604 |
| 69,3859 | 1,57157 | 419,78 | 0,30 | 0,1273 | 00-041-1480, 01-074-1867, 01-083-1604 |
| 70,9601 | 1,54114 | 14279,82 | 10,31 | 0,1714 | 01-074-1867, 01-083-1604, 01-089-8934 |
| 71,7816 | 1,52583 | 3147,16 | 2,27 | 0,1915 | 01-078-4614, 01-083-1604, 04-025-4485 |
| 72,2356 | 1,51753 | 1447,25 | 1,04 | 0,1722 | 01-074-1867, 01-078-4614 |
| 72,6914 | 1,50932 | 1459,19 | 1,05 | 0,2010 | 01-078-4614, 01-083-1604, 04-025-4485 |
| 73,3626 | 1,49742 | 302,08 | 0,22 | 0,0879 | 00-041-1480, 01-083-1604 |
| 73,5126 | 1,49480 | 1091,59 | 0,79 | 0,0780 | 01-083-1604, 04-025-4485 |
| 74,7118 | 1,47422 | 876,26 | 0,63 | 0,1994 | 01-078-4614, 01-083-1604 |
| 76,0080 | 1,45279 | 1524,10 | 1,10 | 0,1848 | 00-041-1480, 01-083-1604, 01-089-8934, 04-025-4485 |
| 76,7219 | 1,44132 | 2922,12 | 2,11 | 0,2077 | 01-074-1867, 01-078-4614, 01-083-1604 |
| 78,0081 | 1,42126 | 2188,39 | 1,58 | 0,2207 | 01-074-1867, 01-078-4614, 01-083-1604, 01-089-8934 |
| 80,6837 | 1,38180 | 6257,03 | 4,52 | 0,1725 | 00-041-1480, 01-089-8934 |
| 81,2118 | 1,37436 | 6577,51 | 4,75 | 0,1863 | 01-074-1867, 01-089-8934 |
| 81,4136 | 1,37154 | 3991,11 | 2,88 | 0,1419 | 00-041-1480, 01-074-1867, 01-089-8934 |

| | | | | | |
|---|---|---|---|---|---|
| 82,4387 | 1,35748 | 700,79 | 0,51 | 0,1408 | 01-074-1867, 01-078-4614 |
| 83,7961 | 1,33947 | 1055,17 | 0,76 | 0,2266 | 01-074-1867, 01-078-4614 |
| 87,1775 | 1,29737 | 1516,57 | 1,09 | 0,2361 | 01-078-4614, 04-025-4485 |
| 88,0006 | 1,28768 | 1989,76 | 1,44 | 0,1950 | 01-089-8934 |

## *Pattern List:*

| Ref.Code | Compound Name | Mineral Name | Chem. Formula | SemiQuant[%] |
|---|---|---|---|---|
| 00-041-1480 | Sodium Calcium Aluminum Silicate | Albite | ( Na , Ca ) Al ( Si , Al )3 O8 | 5 |
| 01-074-1867 | Calcium Carbonate | Vaterite, syn | Ca ( C O3 ) | Stopy možné |
| 01-078-4614 | Calcium Carbonate | Calcite, syn | Ca ( C O3 ) | 40 |
| 01-083-1604 | Potassium Aluminum Silicate | Microcline | K ( Al Si3 O8 ) | 4 |
| 01-089-8934 | Silicon Oxide | Quartz | Si O2 | 50 |
| 04-025-4485 | Magnesium Aluminum Carbonate Hydroxide Hydrate | Quintinite | Mg2 Al ( C O3 )0.5 ( O H )6 ( H2 O )1.5 | 1 |

Oproti vzorku 8 je patrný výrazný nárůst obsahu CaCO3.
