# Supplementary material for: Corrosion Response of Steel to Penetration of Chlorides in DC-Treated Hardened Portland Cement Mortar
Source: Materials (Basel). 2025 Jul 17;18(14):3365. doi: 10.3390/ma18143365 (PMC12300289; doi:10.3390/ma18143365)
Supplement: Supplementary file 1 [file materials-18-03365-s001.zip › protocol s6.pdf]

Protocol S6

Measurement Conditions:

Dataset Name	XADS20_5-90_T120_s6_sp0_rp0_MA-21
File name	\\share\rentgenka980\PC2_XPERT\2024\Kouril\2024-04-23\XADS20_5-90_T120_s6_sp0_rp0_MA-21.xrdml
Sample Identification	MA-21
17min50s	
PHD Lower Level = 4.02 (keV), PHD Upper Level = 9.70 (keV)	
Measurement Start Date/Time	02.05.2024 14:52:49
Operator	localadmin
Raw Data Origin	XRD measurement (*.XRDML)
Scan Axis	Gonio
Start Position [°2θ]	5,2147
End Position [°2θ]	90,1567
Step Size [°2θ]	0,0390
Scan Step Time [s]	116,5350
Scan Type	Continuous
PSD Mode	Scanning
PSD Length [°2θ]	3,35
Offset [°2θ]	0,0000
Divergence Slit Type	Fixed
Divergence Slit Size [°]	1,0000
Specimen Length [mm]	20,00
Measurement Temperature [°C]	25,00
Anode Material	Co
Intended Wavelength Type	K-α1
K-α1 [Å]	1,78901
K-α2 [Å]	1,79290
K-β1 [Å]	1,62083
K-β2 [Å]	1,38113
K-β3 [Å]	1,39261
K-A2 / K-A1 Ratio	0,50000
K-Alpha2 Line Shift	0,00000
K Absorption Edge	1,37868
Generator Settings	40 mA, 35 kV
Diffractometer Type	0000000080910230
Diffractometer Number	0
Goniometer Radius [mm]	240,00
Dist. Focus-Diverg. Slit [mm]	100,00
Incident Beam Monochromator	No
Spinning	No
Fast detector	PIXcel1D_1D detector

Main Graphics, Analyze View:

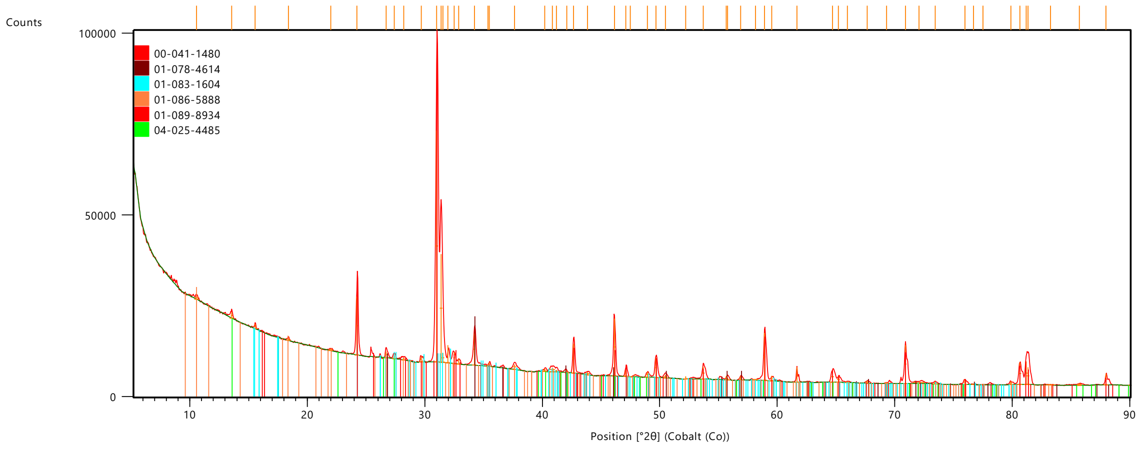

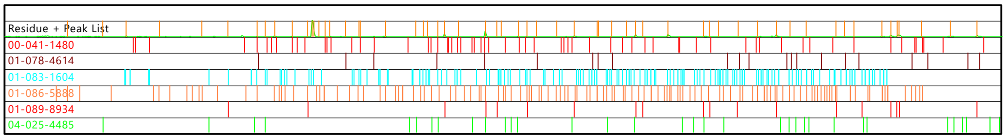

Peak List:

Pos. [°2θ]	d-spacing [Å]	Height [cts]	Rel. Int. [%]	FWHM Left [°2θ]	Matched by
10,5566	9,72355	874,28	1,37	0,2761	01-086-5888
13,5420	7,58689	1559,70	2,45	0,1909	04-025-4485
15,5537	6,61051	1320,57	2,08	0,0981	01-083-1604
18,3839	5,59968	799,79	1,26	0,1284	01-086-5888
21,9874	4,69062	446,84	0,70	0,2760	01-086-5888
24,2264	4,26271	16102,17	25,31	0,1698	01-083-1604, 01-089-8934
26,7229	3,87075	1697,06	2,67	0,2163	00-041-1480, 01-078-4614, 01-083-1604, 01-086-5888
27,3891	3,77833	1118,89	1,76	0,1682	00-041-1480, 01-083-1604, 04-025-4485
28,2020	3,67155	648,54	1,02	0,1658	00-041-1480, 01-086-5888
29,6852	3,49192	1522,55	2,39	0,1172	00-041-1480, 01-086-5888
31,0145	3,34569	63607,42	100,00	0,2244	01-086-5888, 01-089-8934
31,3780	3,30789	29730,82	46,74	0,2318	01-083-1604
31,5390	3,29143	2608,05	4,10	0,2006	01-083-1604
31,9571	3,24947	4923,96	7,74	0,0900	01-086-5888
32,4858	3,19797	3075,72	4,84	0,1226	00-041-1480,

					01-086-5888
32,8937	3,15939	1483,39	2,33	0,0845	00-041-1480
34,2336	3,03922	8703,22	13,68	0,1800	01-078-4614
35,3765	2,94402	399,70	0,63	0,1816	00-041-1480, 01-083-1604, 01-086-5888
35,4947	2,93454	1039,90	1,63	0,0810	00-041-1480, 01-083-1604, 01-086-5888
37,6413	2,77274	1346,99	2,12	0,3355	01-083-1604, 01-086-5888
40,2124	2,60211	751,43	1,18	0,1328	01-086-5888, 04-025-4485
40,8584	2,56269	1168,88	1,84	0,4315	01-086-5888
41,2157	2,54142	520,80	0,82	0,1501	00-041-1480, 01-083-1604
42,1003	2,49037	603,80	0,95	0,0853	00-041-1480, 01-078-4614, 01-083-1604, 01-086-5888, 04-025-4485
42,6578	2,45932	8085,56	12,71	0,1578	01-083-1604, 01-089-8934
43,8554	2,39533	722,35	1,14	0,3728	00-041-1480, 01-083-1604
46,1332	2,28306	15858,19	24,93	0,1315	01-078-4614, 01-086-5888, 01-089-8934, 04-025-4485
47,1167	2,23804	1679,63	2,64	0,1606	01-086-5888, 01-089-8934
47,4776	2,22200	129,70	0,20	0,1632	01-083-1604, 01-086-5888
48,9337	2,15977	1458,37	2,29	0,2476	00-041-1480, 01-083-1604, 04-025-4485
49,6664	2,12988	5341,02	8,40	0,1750	00-041-1480, 01-083-1604, 01-086-5888, 01-089-8934
50,4778	2,09784	850,01	1,34	0,2911	00-041-1480, 01-078-4614
52,2207	2,03250	835,20	1,31	0,0909	01-086-5888
53,7135	1,98004	3376,36	5,31	0,2393	00-041-1480, 01-086-5888, 01-089-8934, 04-025-4485
55,6542	1,91623	1069,88	1,68	0,1196	01-078-4614, 01-083-1604, 01-086-5888

55,7768	1,91235	452,81	0,71	0,1517	01-078-4614, 01-086-5888
56,8993	1,87769	1085,04	1,71	0,2271	00-041-1480, 01-078-4614, 01-083-1604, 01-086-5888
58,1450	1,84086	157,08	0,25	0,5037	01-083-1604, 01-086-5888
58,9249	1,81864	13065,34	20,54	0,1654	01-083-1604, 01-089-8934, 04-025-4485
59,5419	1,80150	1365,95	2,15	0,1158	00-041-1480, 01-083-1604, 01-086-5888, 01-089-8934
61,6828	1,74482	4076,08	6,41	0,1014	01-083-1604, 01-086-5888
64,7206	1,67122	3202,37	5,03	0,2349	01-083-1604, 01-086-5888, 01-089-8934, 04-025-4485
65,2255	1,65969	1500,41	2,36	0,2391	00-041-1480, 01-083-1604, 01-086-5888, 01-089-8934
65,9797	1,64283	236,50	0,37	0,3297	01-083-1604
67,6510	1,60690	652,03	1,03	0,2401	01-078-4614, 01-089-8934
69,2989	1,57330	274,24	0,43	0,2073	00-041-1480, 01-083-1604, 01-086-5888
70,9231	1,54183	10555,19	16,59	0,1649	01-083-1604, 01-086-5888, 01-089-8934
72,0900	1,52018	466,90	0,73	0,9996	01-078-4614, 01-083-1604, 01-086-5888, 04-025-4485
73,4340	1,49617	617,26	0,97	0,2236	01-083-1604, 01-086-5888, 04-025-4485
75,9660	1,45347	1340,21	2,11	0,2131	00-041-1480, 01-083-1604, 01-086-5888, 01-089-8934, 04-025-4485
76,6919	1,44180	214,15	0,34	0,0780	01-078-4614, 01-083-1604
77,5088	1,42896	156,23	0,25	0,1170	00-041-1480, 01-083-1604,

					01-086-5888
79,8760	1,39340	945,31	1,49	0,1806	01-083-1604, 01-086-5888
80,6650	1,38207	5913,95	9,30	0,1946	00-041-1480, 01-089-8934
81,1896	1,37467	6395,49	10,05	0,2113	01-089-8934
81,3545	1,37237	4746,53	7,46	0,2382	00-041-1480, 01-089-8934
83,2772	1,34627	88,24	0,14	0,7567	00-041-1480, 01-086-5888
85,7063	1,31521	403,30	0,63	0,3611	04-025-4485
88,0019	1,28767	3140,83	4,94	0,1768	01-078-4614, 01-089-8934

Pattern List:

Ref.Code	Compound Name	Mineral Name	Chem. Formula	SemiQuant[%]
00-041-1480	Sodium Calcium Aluminum Silicate	Albite	(Na , Ca) Al (Si , Al)3 O8	7
01-078-4614	Calcium Carbonate	Calcite, syn	Ca (C O3)	10
01-083-1604	Potassium Aluminum Silicate	Microcline	K (Al Si3 O8)	10
01-086-5888	Calcium Aluminum Carbonate Sulfate Hydroxide Hydrate	Ettringite	Ca6 Al2 ((S O4)2.81 (C O3)0.51) (O H)12 (H2 O)24.4	5
01-089-8934	Silicon Oxide	Quartz	Si O2	70
04-025-4485	Magnesium Aluminum Carbonate Hydroxide Hydrate	Quintinite, 2T	Mg2 Al (C O3)0.5 (O H)6 (H2 O)1.5	Stopy možné
