# Supplementary material for: Corrosion Response of Steel to Penetration of Chlorides in DC-Treated Hardened Portland Cement Mortar
Source: Materials (Basel). 2025 Jul 17;18(14):3365. doi: 10.3390/ma18143365 (PMC12300289; doi:10.3390/ma18143365)
Supplement: Supplementary file 1 [file materials-18-03365-s001.zip › protocol s7.pdf]

Protocol S7

Measurement Conditions:

Dataset Name	XADS20_5-90_T120_s6_sp0_rp0_MA-16
File name	\\share\rentgenka980\PC2_XPERT\2024\Kouril\2024-04-23\XADS20_5-90_T120_s6_sp0_rp0_MA-16.xrdml
Sample Identification	MA-16
17min50s	
PHD Lower Level = 4.02 (keV), PHD Upper Level = 9.70 (keV)	
Measurement Start Date/Time	24.04.2024 14:17:36
Operator	localadmin
Raw Data Origin	XRD measurement (*.XRDML)
Scan Axis	Gonio
Start Position [°2θ]	4,8147
End Position [°2θ]	89,7567
Step Size [°2θ]	0,0390
Scan Step Time [s]	116,5350
Scan Type	Continuous
PSD Mode	Scanning
PSD Length [°2θ]	3,35
Offset [°2θ]	0,0000
Divergence Slit Type	Fixed
Divergence Slit Size [°]	1,0000
Specimen Length [mm]	20,00
Measurement Temperature [°C]	25,00
Anode Material	Co
Intended Wavelength Type	K-α1
K-α1 [Å]	1,78901
K-α2 [Å]	1,79290
K-β1 [Å]	1,62083
K-β2 [Å]	1,38113
K-β3 [Å]	1,39261
K-A2 / K-A1 Ratio	0,50000
K-Alpha2 Line Shift	0,00000
K Absorption Edge	1,37868
Generator Settings	40 mA, 35 kV
Diffractometer Type	0000000080910230
Diffractometer Number	0
Goniometer Radius [mm]	240,00
Dist. Focus-Diverg. Slit [mm]	100,00
Incident Beam Monochromator	No
Spinning	No
Fast detector	PIXcel1D_1D detector

Main Graphics, Analyze View:

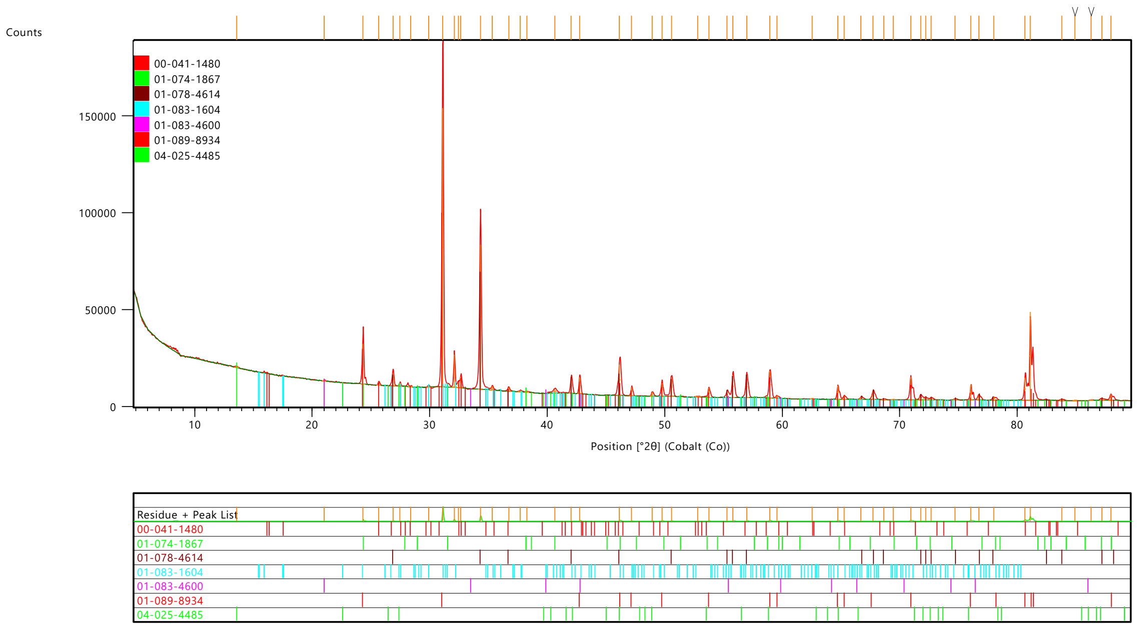

Peak List:

Pos. [°2θ]	d-spacing [Å]	Height [cts]	Rel. Int. [%]	FWHM Left [°2θ]	Matched by
13,5921	7,55907	424,96	0,30	0,2923	04-025-4485
20,9985	4,90885	488,64	0,34	0,0900	01-083-4600
24,3171	4,24706	20755,08	14,42	0,1281	01-074-1867, 01-083-1604, 01-089-8934
25,6535	4,02924	1657,93	1,15	0,0780	00-041-1480
26,8725	3,84960	6252,42	4,35	0,1439	00-041-1480, 01-078-4614
27,4718	3,76718	1784,31	1,24	0,0820	00-041-1480, 01-083-1604, 04-025-4485
28,3910	3,64759	526,44	0,37	0,0854	00-041-1480
29,9247	3,46460	437,80	0,30	0,0780	01-083-1604
31,0883	3,33794	143886,30	100,00	0,1256	01-083-1604, 01-089-8934
32,1024	3,23514	16759,03	11,65	0,0892	01-083-1604
32,4318	3,20315	2233,47	1,55	0,1552	00-041-1480
32,6401	3,18326	5617,25	3,90	0,1330	00-041-1480
34,3154	3,03219	74662,74	51,89	0,1378	01-078-4614
35,3030	2,94995	2016,34	1,40	0,1230	01-083-1604
36,7231	2,83959	1804,30	1,25	0,1605	00-041-1480, 01-078-4614
37,7121	2,76772	609,45	0,42	0,1861	01-083-1604
38,2590	2,72961	395,94	0,28	0,1497	01-074-1867

40,6445	2,57560	1801,76	1,25	0,3237	01-074-1867, 01-083-1604
42,0344	2,49410	7323,26	5,09	0,1608	00-041-1480, 01-078-4614, 01-083-1604, 04-025-4485
42,7614	2,45363	8334,79	5,79	0,1313	01-083-1604, 01-083-4600, 01-089-8934
46,1418	2,28266	15842,17	11,01	0,1968	01-074-1867, 01-078-4614, 01-089-8934, 04-025-4485
47,1857	2,23495	4432,73	3,08	0,1286	01-083-1604, 01-089-8934
48,9233	2,16020	2266,43	1,58	0,1430	00-041-1480, 01-083-1604, 04-025-4485
49,7584	2,12620	6717,15	4,67	0,1468	01-074-1867, 01-083-1604, 01-089-8934
50,5639	2,09450	9400,83	6,53	0,1566	01-078-4614
52,7986	2,01182	328,43	0,23	0,5282	00-041-1480
53,7466	1,97891	4724,05	3,28	0,1352	01-074-1867, 01-089-8934
55,3061	1,92733	3189,13	2,22	0,1902	01-078-4614, 01-083-4600
55,7931	1,91184	11448,35	7,96	0,1725	01-078-4614
56,9762	1,87536	11206,82	7,79	0,1734	00-041-1480, 01-078-4614, 01-083-1604
58,9407	1,81820	13857,17	9,63	0,1646	01-089-8934, 04-025-4485
59,5503	1,80127	1573,97	1,09	0,0780	00-041-1480, 01-074-1867, 01-089-8934
62,5607	1,72277	225,65	0,16	0,1410	00-041-1480, 01-083-1604
64,7202	1,67123	6846,55	4,76	0,1455	01-074-1867, 01-083-1604, 01-089-8934, 04-025-4485
65,2636	1,65883	1790,51	1,24	0,1751	00-041-1480, 01-083-1604, 01-089-8934
66,6943	1,62723	1153,44	0,80	0,1655	01-078-4614, 01-083-1604
67,7369	1,60510	4025,47	2,80	0,2163	01-078-4614, 01-089-8934
68,6287	1,58675	382,47	0,27	0,1876	01-078-4614

69,4628	1,57005	160,38	0,11	0,0780	00-041-1480, 01-074-1867, 01-083-1604
70,9478	1,54137	11606,35	8,07	0,1534	01-083-1604, 01-089-8934
71,7762	1,52593	2459,22	1,71	0,2056	01-078-4614
72,2033	1,51812	1481,69	1,03	0,1639	01-078-4614, 01-083-1604, 04-025-4485
72,6738	1,50963	1219,96	0,85	0,1811	01-074-1867, 01-078-4614, 01-083-1604, 04-025-4485
74,6945	1,47451	887,41	0,62	0,1001	01-078-4614, 01-083-1604
76,0450	1,45219	7946,14	5,52	0,1260	01-083-1604, 01-089-8934, 04-025-4485
76,7215	1,44133	2539,68	1,77	0,2271	01-078-4614
77,9990	1,42140	1741,41	1,21	0,2118	01-074-1867, 01-078-4614
80,6599	1,38214	11565,89	8,04	0,1669	00-041-1480, 01-089-8934
81,1211	1,37563	45544,79	31,65	0,1278	01-089-8934
83,7825	1,33964	848,80	0,59	0,2247	01-078-4614
87,1837	1,29729	1227,94	0,85	0,1352	01-074-1867, 01-078-4614, 04-025-4485
87,9668	1,28808	3120,12	2,17	0,1834	01-074-1867, 01-089-8934

Pattern List:

Ref.Code	Compound Name	Mineral Name	Chem. Formula	SemiQuant[%]
00-041-1480	Sodium Calcium Aluminum Silicate	Albite	(Na , Ca) Al (Si , Al)3 O8	5
01-074-1867	Calcium Carbonate	Vaterite, syn	Ca (C O3)	Stopy možné
01-078-4614	Calcium Carbonate	Calcite, syn	Ca (C O3)	35
01-083-1604	Potassium Aluminum Silicate	Microcline	K (Al Si3 O8)	5
01-083-4600	Calcium Hydroxide	Portlandite, syn	Ca (O H)2	Stopy možné
01-089-8934	Silicon Oxide	Quartz	Si O2	55
04-025-4485	Magnesium Aluminum Carbonate Hydroxide Hydrate	Quintinite	Mg2 Al (C O3)0.5 (O H)6 (H2 O)1.5	stopy

Oproti vzorku 4 je patrný výrazný nárůst obsahu $CaCO_3$.
