# Supplementary figures and images for: Corrosion Response of Steel to Penetration of Chlorides in DC-Treated Hardened Portland Cement Mortar
Source: Materials (Basel). 2025 Jul 17;18(14):3365. doi: 10.3390/ma18143365 (PMC12300289; doi:10.3390/ma18143365)

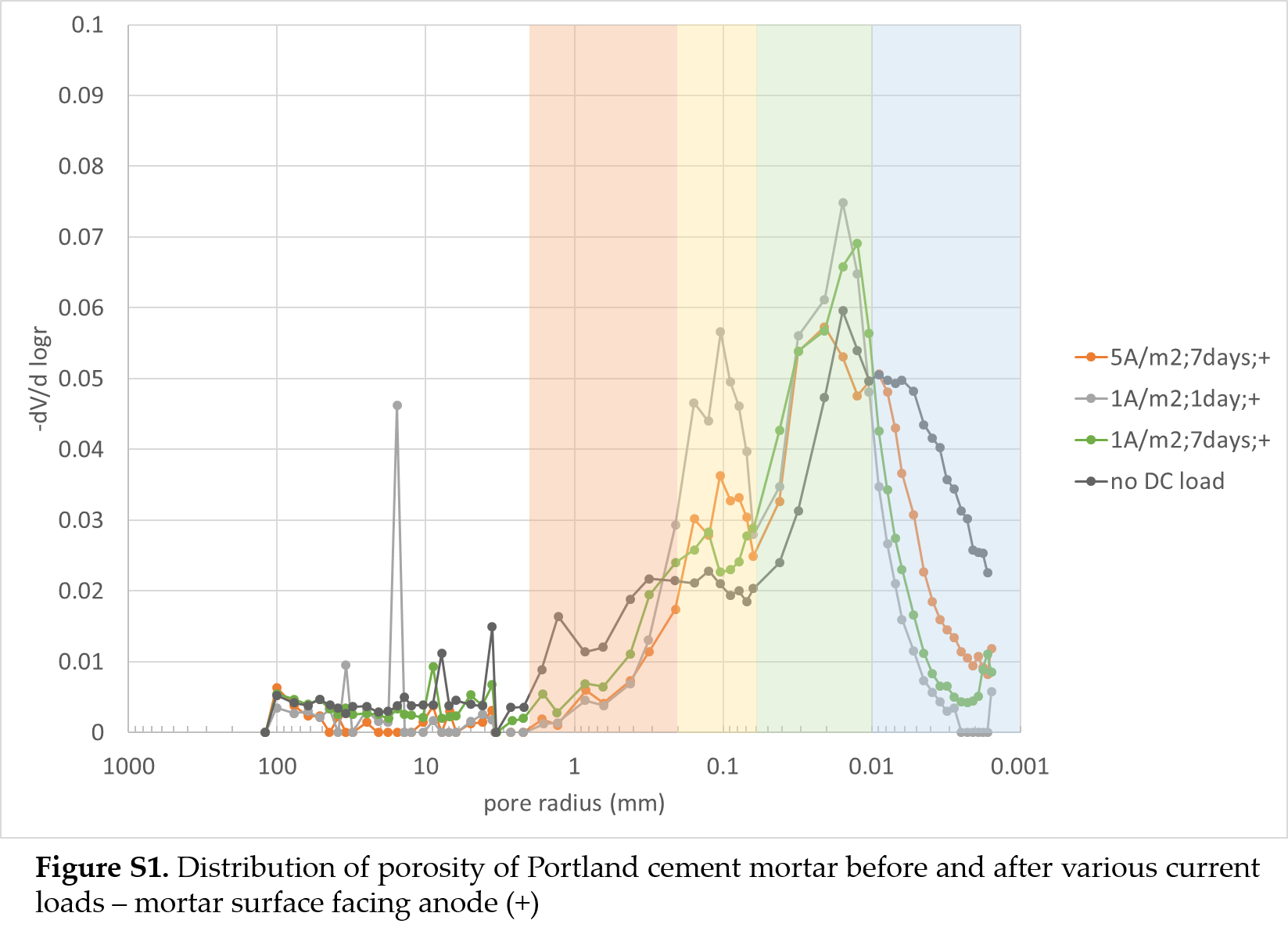

Supplement: Supplementary file 1 [file materials-18-03365-s001.zip › Figure S1.png]

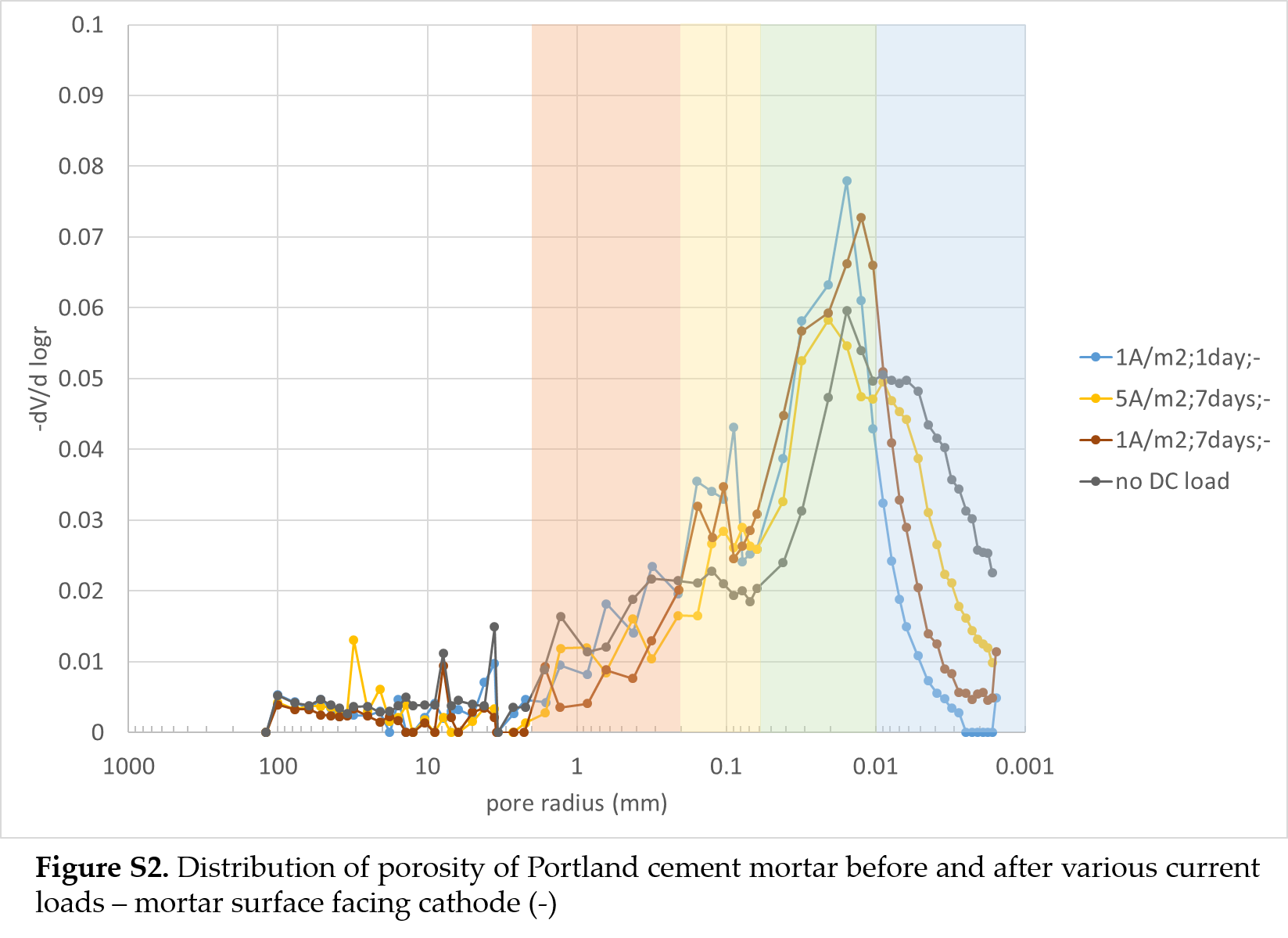

Supplement: Supplementary file 1 [file materials-18-03365-s001.zip › Figure S2.png]
